# A comparison of the composition and functions of the oral and gut microbiotas in Alzheimer’s patients

**DOI:** 10.3389/fcimb.2022.942460

**Published:** 2022-08-24

**Authors:** Lili Chen, Xinhua Xu, Xiaoqi Wu, Huizhen Cao, Xiuli Li, Zhaoyi Hou, Bixia Wang, Jinxiu Liu, Xinli Ji, Ping Zhang, Hong Li

**Affiliations:** ^1^ The School of Nursing, Fujian Medical University, Fuzhou, China; ^2^ Shengli Clinical Medical College of Fujian Medical University, Fuzhou, China; ^3^ Nursing Department, Fujian Provincial Hospital, Fuzhou, China; ^4^ College of Traditional Chinese Medicine, Fujian University of Traditional Chinese Medicine, Fuzhou, China; ^5^ Nursing Department, Fujian Provincial Hospital South Branch, Fuzhou, China

**Keywords:** Alzheimer’s disease, gut microbiome, oral microbiome, 16S ribosomal RNA, oral-gut-brain axis

## Abstract

**Objective:**

Alterations in the oral or gut microbiotas have been reported in patients with subjective and mild cognitive impairment or AD dementia. However, whether these microbiotas change with the severity of the AD spectrum (mild, moderate, and severe AD) remains unknown. Thus, we compared alterations in the composition and gene functions of the oral and gut microbiota between different phases of AD.

**Methods:**

We recruited 172 individuals and classified these into three groups: healthy controls (n = 40), a mild AD group (n = 43) and a moderate AD group (n = 89). Subgingival plaques and fecal samples were collected from all individuals. Then, we conducted 16S ribosomal RNA. sequencing to analyze the microbiotas.

**Results:**

In order of the severity of cognition impairment (from normal to mild and to moderate AD), the oral abundances of the phyla Firmicutes and Fusobacteria showed a gradual upwards trend, while the abundance of the Proteobacteria phylum gradually decreased. In contrast, the abundance of the Firmicutes and Bacteroidetes phyla in the gut decreased progressively, while that of the Proteobacteria, Verrucomicrobia and Actinobacteria phyla increased gradually. Key differences were identified in the microbiomes when compared between the mild AD and moderate AD groups when applying the linear discriminant analysis effect size (LEfSe) algorithm. LEfSe analysis revealed alterations that were similar to those described above; furthermore, different bacterial taxa were associated with MMSE scores and age. KEGG analysis showed that the functional pathways associated with the oral microbiota were mainly involved in membrane transport and carbohydrate metabolism, while the gene functions of the fecal microbiota related to metabolism of amino acids, energy, cofactors and vitamins; identified significant differences among the three groups. Venn diagram analysis revealed that the number of genera that were present in both the oral and gut microbiota increased progressively from NC to mild AD and then to moderate AD.

**Conclusions:**

This study is the first to report a comparative analysis of the oral and fecal microbiota of patients with mild and moderate AD. The compositions and functions of the oral and gut microbiotas differed when compared between different stages of AD.

## Introduction

Alzheimer’s disease (AD) is a neurodegenerative disorder characterized by a slow and progressive memory decline and cognitive dysfunction (Zhao, 2020). Over the last few decades, the prevalence of AD has been rapidly increasing due to a global rise in life expectancy (Zhao, 2020). Approximately 50 million people suffer from dementia, and this number is projected to increase to 152 million by 2050, rising particularly in low-income and middle-income countries that already account for approximately two-thirds of the people with dementia worldwide (International, Alzheimer’s Disease, and McGill University. World Alzheimer Report, 2021). However, no preventative or disease-modifying treatments are available for AD (Honig et al., 2018). Dementia affects people, their families, and the economy; furthermore, the global cost of AD is estimated to be approximately US$1 trillion annually (Alzheimer’s Disease International and Patterson, 2018).

By focusing on the nervous system, only limited progress has been made with regards to the Limited progress on the etiopathology of AD. The etiology of AD is likely to involve an interplay between genetic and environmental factors and the central nervous system (CNS) or systemic inflammation (Lourida et al., 2019). Researchers have suggested that microbiome dysregulation plays an important role in the pathogenesis of AD (Cryan et al., 2019; Park et al., 2021). The gastrointestinal tract and the oral cavity are the main sites for the distribution of symbiotic microorganisms in the human body (Ogobuiro et al., 2021). Research on oral microbiota has attracted considerable attention over the last decade, and several studies have identified alterations in the oral microbiotas of patients with AD (Liu et al., 2019; Na et al., 2020; Wu et al., 2021). Oral microbiotas may be associated with AD owing to the short route to the brain through the cranial nerves. At autopsy, oral bacteria have been found in the brain tissue and cerebrospinal fluid of patients with histopathologically confirmed AD (Miklossy, 1994; Riviere et al., 2002; Dominy et al., 2019). The density of these oral bacteria was approximately seven times higher, and their diversity far greater, in the brains of patients with AD than in cognitively normal individuals (Miklossy, 2008). These findings suggest that oral microbiome disorders may increase infections by opportunistic pathogens in the brain of patients, thereby contributing to AD development. The human intestinal tract harbors a complex community of microbes accounting for the vast majority of the resident microbial population (Human Microbiome Project, 2012). Studies on the gut–brain axis have highlighted the potential impacts of the gut microbiota on the brain through a bidirectional communication system that is connected *via* neural, immunity-related, endocrinological, and metabolic pathways (Cryan et al., 2019). Several studies have reported patients with AD with altered gut microbiotas, thus suggesting that these changes may be involved in the pathogenesis of AD (Vogt et al., 2017; Zhuang et al., 2018; Sochocka et al., 2019; Bonnechère et al., 2020; Ling et al., 2020).

Many studies have shown that oral and gut microbiotas may be associated with the occurrence and development of AD (Li et al., 2019; Liu et al., 2019; Guo et al., 2021; Sheng et al., 2021). The progression of Alzheimer’s disease, referred to as the Alzheimer’s disease continuum, consists of three phases: preclinical Alzheimer’s disease, mild cognitive impairment (MCI) due to AD, and dementia due to AD. The Alzheimer’s dementia phase can be further broken down into the stages of mild, moderate, and severe AD, which reflect the degree to which symptoms interfere with a patient’s ability to carry out everyday activities (Marasco, 2020). Based on prior research findings, two important questions remain: The first is whether dysbiosis of the microbiome varies with the severity of the AD phase. Studies have revealed oral or gut microbiome changes in different stages of the Alzheimer’s disease continuum. Subjective cognitive decline (SCD) is the earliest symptomatic manifestation of preclinical Alzheimer’s disease. Holmer et al. (2021) compared the subgingival microbiotas of people with AD, MCI, and SCD with those of cognitively healthy individuals, revealing higher microbial richness and evenness in cases than in controls; these authors also reported differences across the four groups. Sheng et al. (2021) showed that the composition of the gut microbiota was different in individuals with SCD. Liu et al. (2019) also showed that the gut microbiome was different between healthy controls and patients with MCI or AD. Microbiomes can help distinguish healthy controls from patients with MCI or AD, or patients with AD from patients with MCI. Li et al. (2019) showed that patients with MCI had gut microbiomes that were similar to those of patients with AD. Guo et al. (2021) further showed that the degree of gut dysbiosis worsened with the progression of the disease from MCI to AD. However, whether the dysbiosis of the microbiome varies with the severity of the AD phase (e.g., mild, moderate, and severe AD) remains unknown.

The second question pertains to the association between the oral and gut microbiotas in patients with AD. The oral cavity initiates the gastrointestinal tract. The oral and gut microbiotas differ, especially in terms of their resident bacteria, although bacteria from these locations may interact because the alimentary tract is a continuous tube running from the oronasal cavity to the anus (Maki et al., 2021). Associations between these two microbial communities have been extensively investigated. Iwauchi et al. (2019) reported that the transition of subgingival plaque bacteria to the gut was more prevalent in older adults than in younger adults. The average person swallows more than 1000 mL of saliva per day, almost all of which enters the gastrointestinal tract (Said et al., 2014). Considering the decline in functionality of the gastrointestinal tract in the elderly, a higher number of oral bacteria may reach the gut than in healthy adults owing to diminished extinction by gastric juices and/or bile acid. Given that AD is a major age-related neurodegenerative disorder, we hypothesized that the transition of oral bacteria to the gastrointestinal tract perhaps becomes more severe as AD progresses. The oral microorganisms that enter the gastrointestinal tract, to a certain extent, change the structure of the intestinal microbial community, thus leading to metabolic endotoxins, which further induce inflammation-related changes in various tissues and organs (Narengaowa et al., 2021). Inflammation is a key characteristic of numerous diseases, including neurodegenerative disorders (Paouri and Georgopoulos, 2019). Sequencing of the 16S ribosomal RNA (16S rRNA) gene has facilitated the comparison of the composition of oral and gut microbiotas among individuals with AD and has revealed a correlation between specific microbial communities and AD. However, most previous research on oral and gut microbiomes was conducted separately in an organ-specific manner, rather than in an integrative context. The most recent studies have proven the involvement of the microbiome in inter-organ networks, such as the gut–brain and oral–gut microbiome axes (Cryan et al., 2019; Narengaowa et al., 2021; Park et al., 2021). Therefore, a comparative analysis of the oral and fecal microbiotas of patients with AD might reveal whether there is intestinal colonization of the oral microbiota and whether this modulates pathophysiological processes in patients with AD. Oral and intestinal-specific bacterial species and their products may be potential biomarkers for the prevention and clinical diagnosis of AD (Narengaowa et al., 2021).

In this study, we performed bacterial 16S rRNA gene sequencing with DNA isolated from subgingival plaques and fecal samples of participants to simultaneously detect oral and gut microbial communities. We investigated the composition and functional alterations of the oral and gut microbiotas in individuals with mild or moderate AD, comparing data to those in their sex- and age-matched controls with normal cognition (NC). Finally, we investigated the similarity between the oral and gut microbiotas.

## Materials and methods

### Study participants

We recruited 172 individuals (normal cognition controls, n = 40; mild AD dementia, n = 43; and moderate AD dementia, n = 89) from the Memory Clinic in Fujian Provincial Hospital (Fujian, China) between February 2020 and February 2022. Our study was part of a multicenter-based longitudinal observational study in China that focused predominantly on individuals with AD dementia and included cognitively normal subjects as controls. All patients were evaluated with a complete medical history evaluation, physical examination, neurological, and neuropsychological assessment, neuroimaging (magnetic resonance imaging), and clinical biochemistry examinations. Blood tests to exclude secondary causes of dementia included a complete blood count, blood chemistry tests, thyroid function tests, homocysteine, vitamin B12/folate, and syphilis serology. Conventional brain MRI or CT scans confirmed the absence of structural lesions such as brain tumors, traumatic brain injuries, hydrocephalus, or severe white matter diseases.If routine diagnostic work-up was unable to identify dementia associated with Alzheimer's disease, then we used apolipoprotein E (APOE) or Presenilin-1 genotyping and amyloid PET in a selective manner based on individual agreement.The clinical assessment and diagnosis of AD dementia were made by experienced neurologists and memory clinic specialists independent from the study and according to the guidelines for dementia due to AD proposed by the National Institute on Ageing-Alzheimer’s Association (NIA-AA) workgroups and the criteria of the Diagnostic and Statistical Manual (DSM)-V (Dubois et al., 2014). Healthy controls were recruited in the same proportions according to gender and age population; most of these were the spouses of the patients who had lived in the same household and been on the same diet together for at least 20 years. The healthy controls had CDR scores of 0 (no dementia), and had no significant memory complaints. To eliminate the influence of regional lifestyles on the oral and gut microbial communities (Sandhu et al., 2017; Zhu et al., 2020), all participants were community-dwelling Han individuals aged 60 to 90 years-of-age and who were ordinary residents of Fuzhou. Each participant completed a lifestyle questionnaire with detailed demographic and medical history data (such as hypertension and diabetes mellitus statuses) and a neuropsychological assessment including the Chinese version of the Mini-Mental State Examination (MMSE); see **Table 1** and **Supplementary Table 1**. Moreover, memory clinic specialists used the Clinical Dementia Rating (CDR) scale to evaluate the severity of AD dementia. Patients with AD and a total CDR score of 1 were diagnosed as suffering from mild AD dementia and those with CDR score of 2 were diagnosed as having moderate AD dementia (Morris, 1997; O'Bryant et al., 2008).

**Table 1:** General characteristics of participants.

Characteristics	CN (*n* = 40)	mild AD (*n* = 43)	moderate AD (*n* = 89)	*p*-value	CN vs. mild AD	CN vs. moderate AD	mild AD vs. moderate AD
					*p*_1_	*p*_2_	*p*_3_
Age, years medians (IQR)	70.00 (59.25–74.00)	79.00 (73.00–85.00)	82.00 (75.50–86.50)	<0.001**	<0.001**	<0.001**	0.164
BMI, kg/m², medians (IQR)	21.91 (20.92–25.24)	22.20 (20.60–23.87)	22.60 (21.40–24.20)	0.652	–	–	–
Education, medians (IQR)	8 (5–8)	11 (8–13)	9 (5–12)	0.001**	0.001**	0.006**	0.628
MMSE score, medians (IQR)	26 (25–28)	22 (21–23)	18 (17–19)	<0.001**	0.009**	<0.001**	<0.001**
Gender, M/F	16/24	15/28	33/56	0.890	0.630	0.752	0.806
Smoking, *n* (%)	6 (17.14%)	7 (26.92%)	6 (21.43%)	0.654	0.356	0.667	0.637
Drinking, *n* (%)	11 (27.50%)	10 (37.04%)	7 (25.00%)	0.581	0.409	0.818	0.334
Married, *n* (%)	20 (60.60%)	26 (63.40%)	47 (56.00%)	0.710	0.804	0.647	0.427

Age, BMI, education and MMSE scores are expressed as medians (IQR). Gender, smoking, drinking and married are expressed as number of individuals (%). p-values among the three groups were calculated using a Kruskal-Wallis test. p_1_, p_2_, and p_3_ were adjusted for significance with Bonferroni correction for multiple tests. NC, normal cognition controls; mild AD, mild Alzheimer’s disease; moderate AD, moderate Alzheimer’s disease; BMI, body mass index; MMSE, Mini-Mental State Examination; M, male; F, female; IQR, interquartile range.

The exclusion criteria included: 1) other causes or types of dementia; 2) a family history of dementia; 3) any other neurodegenerative disease, such as Parkinson’s disease; 4) confirmed mental illness, such as schizophrenia; 5) severe cardiac, pulmonary, hepatic, or renal disease, or any tumor; 6) intestinal diseases, such as irritable bowel syndrome; 7) the intake of antibiotics, glucocorticoids, or probiotics within the previous month; 8) known active viral, bacterial, or fungal infections, or autoimmune diseases; and 9) severe auditory, visual or motor deficits that might interfere with cognitive testing. The participants also underwent an oral health status check within the two months prior to the study to exclude individuals with the following conditions: oral surgery or dental procedures, inflammation of oral or perioral tissues, and other oral cavity chronic diseases. We did not exclude individuals with periodontal diseases and did not systematically evaluate the oral and dental conditions of the participants. However, they were subject to the Kayser–Jones Brief Oral Health Status Examination (BOHSE) (Kayser-Jones et al., 1995).

The Ethics Committee of Fujian Provincial Hospital approved the study protocol (reference number: K2020-09-025) and we obtained written and informed consent from each individual or their spouses before enrollment.

### Sample handling and collection

Following enrolment, we performed visual dental inspections to determine the oral status of all individuals. After the periodontal examination, the deepest or the most representative periodontal pocket was selected for subgingival microbial sampling. A trained dentist collected subgingival plaques from the subgingival surfaces of the teeth using periodontal curettes (Graceycurett, Hu-Friedy, USA) (Cockburn et al., 2012; Haririan et al., 2014). No samples were collected from patients without teeth or those with dental implants. All subgingival plaque samples were stored at −80°C until further processing. Each participant was asked to collect a fresh fecal sample in the morning. Several community-dwelling older individuals could not send their samples to the hospital immediately; these subjects were given fecal collection containers (SARSTEDT, Germany) containing approximately 5 ml of special cytoprotective agents to preserve the DNA in the stool at room temperature for 10–14 days until the fecal samples could be transferred to the laboratory for storage at -80°C prior to processing.

### DNA extraction and 16S rRNA gene amplicon sequencing

Oral and gut DNA samples were processed for Deoxyribonucleic acid (DNA) extraction, Polymerase chain reaction (PCR) amplification, and sequencing of the V3–V4 hypervariable regions of the bacterial 16S rRNA gene at the DNA Sequencing and Genomics Laboratory of Sangon BioTech (Shanghai). Total community genomic DNA extractions were performed using an E.Z.N.A. Soil DNA Kit (Omega, USA) following the manufacturer’s instructions. PCRs were initiated immediately after extracting the DNA samples. The 16S rRNA V3–V4 fragment was amplified using KAPA HiFi Hot Start Ready Mix (2×) (TaKaRa Bio, Japan). Two universal bacterial 16S rRNA gene amplicon PCR primers (Polyacrylamide gel electrophoresis (PAGE)-purified) were used: a forward primer (CCTACGGGNGGCWGCAG), and a reverse primer (GACTACHVGGGTATCTAATCC). The PCR program was run in a thermocycler (Applied Biosystems 9700, USA) with the following cycling conditions: 1 cycle of denaturation at 95°C for 3 min, 5 cycles of denaturation at 95°C for 30 s, annealing at 45°C for 30 s, and elongation at 72°C for 30 s. This was followed by 20 cycles of denaturation at 95°C for 30 s, annealing at 55°C for 30 s, elongation at 72°C for 30s, and a final extension at 72°C for 5 min. The PCR products were checked by electrophoresis in 1% (w/v) agarose gels in TBE buffer (Tris, boric acid, EDTA), stained with ethidium bromide (EB) and visualized under UV light.

Sequencing was performed using the Illumina MiSeq system (Illumina MiSeq, USA). The raw sequencing reads were detected to remove the primer region and low-quality sequences. Chimera sequences arising from the PCR amplification products were detected and excluded using Mothur (http://www.mothur.org) based on the GreenGenes database. High-quality reads, reaching 97% nucleotide similarity, were clustered into operational taxonomic units (OTUs) according to the algorithm in the Ribosomal Database Project (RDP) database. We constructed summaries of the taxonomic distributions of OTUs using these taxonomies and used these summaries to calculate the relative abundances of microbiota at the phylum, class, order, family, and genus levels. Furthermore, we performed the Pan/Core OTU species analysis of the oral and gut microbiomes, and found that the samples were sufficient for this study (**Supplementary Figure 1**).

### Bioinformatic analysis

We used Quantitative Insights into Microbial Ecology 2 (QIIME2) (Bolyen et al., 2019) and R software to conduct analyses of α diversity (Shannon, Simpson, observed OTUs, Chao1 and ACE indices) and β diversity [Bray–Curtis dissimilarity, and principal coordinate analysis (PCoA)]. Permutational multivariate analysis of variance (PERMANOVA) was employed to identify the different microbial communities among groups. We performed Kruskal–Wallis tests to identify significant alterations of the predominant oral and gut microbiotas among the three groups at the different levels. Then, we performed *post hoc* group comparisons by applying the Bonferroni adjustment as appropriate. The key taxa responsible for the differences in the subgingival and fecal microbiotas between the mild and the moderate AD groups were identified by using the LEfSe (Linear discriminant analysis Effect Size) algorithm for biomarker discovery, which emphasizes both statistical significance and biological relevance (Segata et al., 2011). We defined a significant α of 0.05 and an effect size threshold of 2 for all biomarkers discussed in this study. To determine the associations between the differential bacterial taxa identified with the LEfSe algorithm between the two AD groups, and clinical indicators such as their age, years of education, BMI, and MMSE scores, we performed correlation analysis using Spearman’s rank correlation. We applied a Phylogenetic Investigation of Communities by Reconstruction of Unobserved States (PICRUSt) algorithm to detect the predicted functions in the microbial communities. The predicted functional genera were categorized according to the Kyoto Encyclopedia of Genes and Genome (KEGG) orthology (KO). Because we characterized gut and oral microbiota samples from the same individuals, we tried to construct a Venn diagram to explore the similarity between the two microbial communities within patients.

### Statistical analysis

Results are presented as numbers with percentages, means with standard deviation (mean ± SD), or medians with interquartile ranges (medians, IQR). We applied Student’s *t* or Mann–Whitney *U* tests for two groups and one-way Analysis of Variance (ANOVAs) or Kruskal–Wallis tests for more than two groups to determine statistical significance. Then, we evaluated *post hoc* group comparisons by applying the Bonferroni adjustment as appropriate. Pearson’s chi-squared test or Fisher’s exact test were performed to compare categorical variables. We performed all statistical analyses using IBM SPSS Statistics 26.0 and R-3.6.3 software (Sheng et al., 2021). The R package and GraphPad Prism v6.0 were used to generate graphs. All tests of significance were two sided and *p* < 0.05 was considered statistically significant.

## Results

### Individual characteristics

The general characteristics of individuals in the normal cognition control (n = 40), mild AD (n = 43) and moderate AD (n = 89) groups are shown in **Table 1**. Age, education, and MMSE score differed significantly among the three groups (*p* < 0.001, *p* = 0.001, *p* < 0.001, respectively). Individuals in the mild AD and moderate AD groups were significantly older and had significantly higher education levels than controls with normal cognition. The MMSE scores were significantly higher in the NCs than in the mild AD and moderate AD groups (*p*_1_ = 0.009, *p*_2_ < 0.001, *p*_3_ < 0.001, respectively). Variables such as BMI, gender, smoking, drinking, and marriage status were similar among the three groups (*p* > 0.05).

### α and β diversity

**Figure 1:**
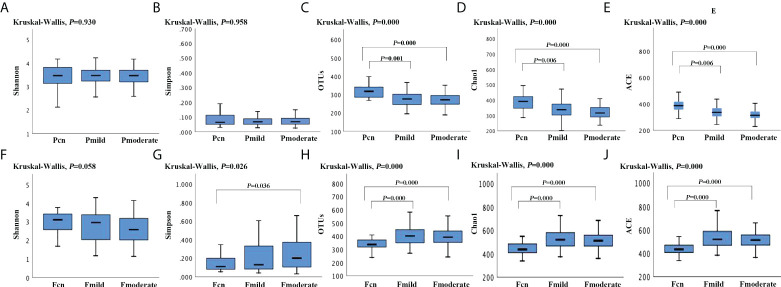
The α-diversity analysis (Shannon, Simpson, the observed OTUs, ACE, Chao-1 index) of the oral and gut microbiotas among the three groups. The oral α-diversity analysis **(A–E)** and the gut α-diversity analysis **(F–J)** are presented. *p*-values calculated using Kruskal–Wallis-test among three groups, and *post hoc* group comparisons evaluated by Bonferroni adjustment. Pcn, subgingival plaque of normal cognition controls; Fcn, feces of normal cognition controls; Pmild, subgingival plaque of mild AD; Fmild, feces of mild AD; Pmoderate, subgingival plaque of moderate AD; Fmoderate, feces of moderate AD.

**Figure 2:**
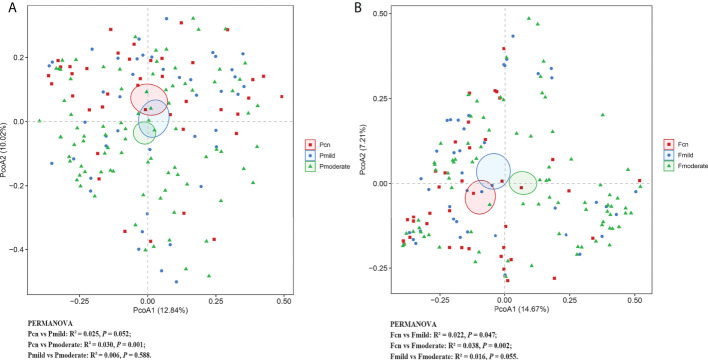
The β-diversity analysis of the subgingival plaque **(A)** and fecal **(B)** microbiota among the three groups. PCoA based on the Bray-Curtis of β-diversity analysis. PERMANOVA tests if the centroids, similar to means, of each group are significantly different from each other. R² statistic showing the community variation between the compared groups with significant *p*-values. Pcn, subgingival plaque of normal cognition controls; Fcn, feces of normal cognition controls; Pmild, subgingival plaque of mild AD; Fmild, feces of mild AD; Pmoderate, subgingival plaque of moderate AD; Fmoderate, feces of moderate AD; PCoA, principal coordinate analysis.

**Figure 1** shows the fecal microbial diversity as estimated using the Simpson Index; this was significantly lower in individuals with moderate AD than in NCs (*p* = 0.036), although we found similar oral microbial diversities in these two groups. The oral microbial richness (the observed OTUs, Chao-1 and ACE index) showed a progressively decreasing prevalence from the NC to the mild AD to the moderate AD groups; notably, these were significantly reduced in patients with mild and moderate AD. In contrast, the richness of the gut microbiota was significantly higher in the mild AD and moderate AD groups than in the NC group. **Figure 2** demonstrates significantly different microbial communities of the gut microbiomes among NC, mild AD and moderate AD groups, as determined by a PCoA plot based on Bray–Curtis dissimilarity (PERMANOVA, Bray–Curtis: NC *vs.* mild AD: R² = 0.022, *p* = 0.047; NC *vs.* moderate AD: R² = 0.038, *p* = 0.002; mild AD *vs.* moderate AD: R² = 0.016, *p* = 0.055). We found a significant difference in the oral microbiomes between the moderate AD and NC groups (PERMANOVA, Bray–Curtis: R² = 0.030, *p* = 0.001).

α diversity analysis revealed a significantly higher diversity (Shannon’s and Simpson’s index) and lower richness (the observed OTUs, ACE, and Chao–1 index) for the oral microbiota than for the gut microbiota in the three groups (**Supplementary Table 2**). PCoA plots based on Bray–Curtis dissimilarity for the microbiota genus in each sample indicated that the oral and gut microbiota formed clearly separate groups (PERMANOVA, Bray–Curtis: *p* < 0.01; **Supplementary Table 2**).

### Alterations of microbiomes in the mild AD, moderate AD and healthy control groups

**Figure 3:**
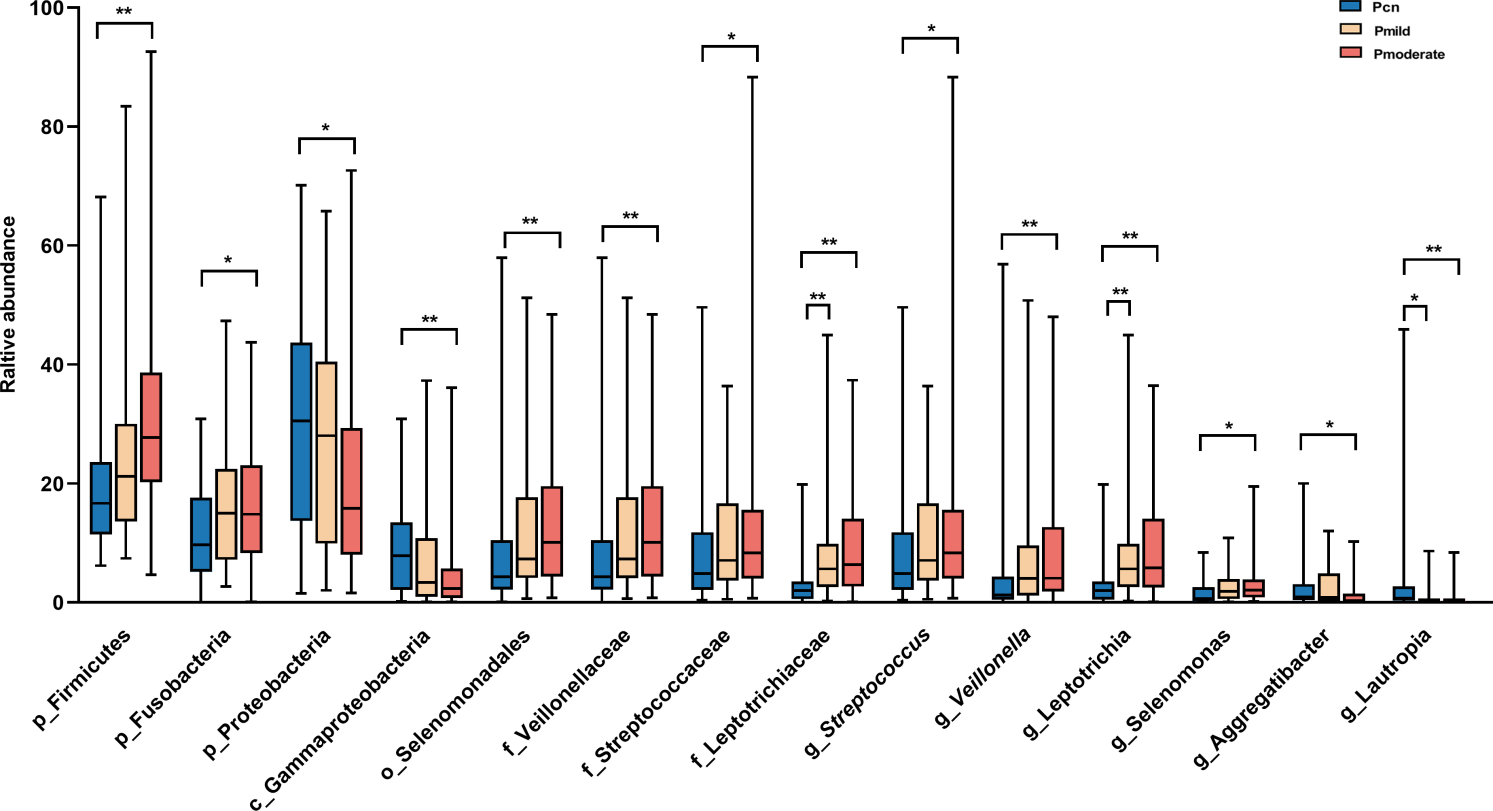
Alteration of oral predominant microbiotas among the three groups. Comparison among the three groups by Kruskal–Wallis-test analysis, and significant taxa classifications obtained by *Post Hoc* test using Bonferroni adjustment. *p <0.05, ** *p*<0.01; Pcn, subgingival plaque of normal cognition controls; Pmild, subgingival plaque of mild AD; Pmoderate, subgingival plaque of moderate AD; IQR, interquartile range.

**Figure 4:**
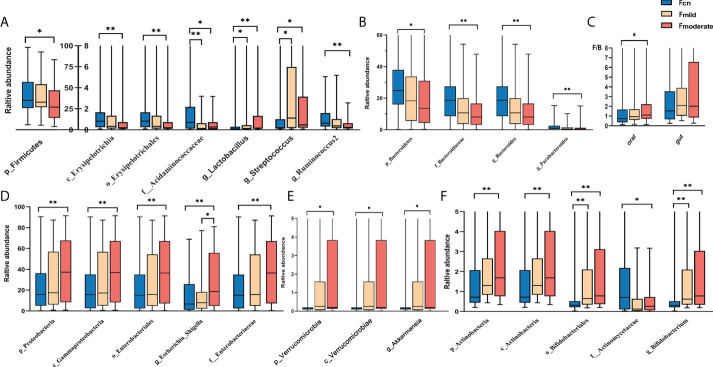
Alteration of gut predominant microbiotas among the three groups **(A–F)**. Comparison among the three groups by Kruskal–Wallis-test analysis, and significant taxa classifications obtained by *Post Hoc* test using Bonferroni adjustment. *p <0.05, ** *p*<0.01; Fcn, feces of normal cognition controls; Fmild, feces of mild AD; Fmoderate, feces of moderate AD; IQR, interquartile range.

We found significant alterations of the predominant oral and gut microbiotas among the three groups at the phylum, class, order, family, and genus levels (**Figures 3**, **4**). The predominant microbiotas were identified using the microbial taxa with average relative abundances greater than 1% in any of the three groups. For the oral microbiomes, the phylum Firmicutes and its corresponding order Selenomonadales, the family Veillonellaceae, the family Streptococcaceae, the genus *Selenomonas*, the genus *Veillonella*, the genus *Streptococcu*, and the phylum Fusobacteria and its corresponding family Leptotrichiaceae, the genus *Leptotrichia*, showed a progressively increased prevalence from NC to mild AD and to moderate AD groups. In particular, the abundances of these taxa were significantly higher in the moderate AD group than in the NC group (*p* < 0.05). Notably, the abundances of the family Leptotrichiaceae and genus *Leptotrichia* of the Fusobacteriia phylum were significantly higher in the mild AD group than in the NC group (*p* = 0.002, *p* = 0.002). The phylum Proteobacteria and its corresponding class Gammaproteobacteria, the genus *Aggregatibacter*, and the genus *Lautropia*, showed a trend towards a progressive decrease from the NC to the mild AD and the moderate AD groups; these taxa were significantly less abundant in the moderate AD group than in the NC group (*p* < 0.05). Notably, the genus *Lautropia* was less abundant in the mild AD group than in the NC group. There was a trend towards a progressive increase in the ratio of Firmicutes to Bacteroidetes (F/B ratio) in oral bacteria from the mild AD to the moderate AD groups as compared to the ratio in the NC group. In particular, this ratio was significantly higher in the moderate AD group than in the NC group (*p =* 0.036) (**Figure 4C**).

**Figure 4** shows diametrically opposite abundance trends for the gut and oral microbiotas. The phylum Firmicutes and its corresponding class Erysipelotrichia, the order Erysipelotrichales, the family Acidaminococcaceae, the genus *Ruminococcus2*, the genus *Phascolarctobacterium*, and the genus *Clostridium_IV*, demonstrated a markedly lower abundance in the moderate AD group than in NC individuals (*p* < 0.05). Intriguingly, the abundances of the cariogenic dental pathogens (the *Lactobacillus* (*p* = 0.023, *p* < 0.001) and *Streptococcus* (*p* = 0.012, *p* = 0.020) genera) were also higher in the mild and moderate AD and groups than in the NC group (**Figure 4A**). In addition, the phylum Bacteroidetes and its corresponding family Bacteroidaceae, genus *Bacteroides*, along with the genus *Parabacteroides*, presented a trend towards a progressively decreased abundance from the NC group to the mild AD group and the moderate AD group; these taxa showed significantly lower abundances in the moderate AD group than in the NC group (*p* < 0.05) (**Figure 4B**). However, the phylum Proteobacteria and its corresponding class Gammaproteobacteria, the order Enterobacteriales, the family Enterobacteriaceae, the genus *Escherichia_Shigella*, and the phylum Verrucomicrobia and its corresponding class Verrucomicrobiae and genus *Akkermansia*, and the phylum Actinobacteria and its corresponding class Actinobacteria, the order Bifidobacteriales, the family Actinomycetaceae, and the genus *Bifidobacterium* showed progressively higher prevalence from the mild AD to the moderate AD groups as compared to those in the NC group; these taxa were more abundant in patients with moderate AD than in NC individuals (**Figures 4D–F**). Moreover, the abundance of phylum Actinobacteria and its corresponding class Actinobacteria, along with the order Bifidobacteriales, was also significantly higher in the mild AD group than in the NC group.

### The identification of crucial bacteria to differentiate between patients with mild and moderate AD

**Figure 5:**
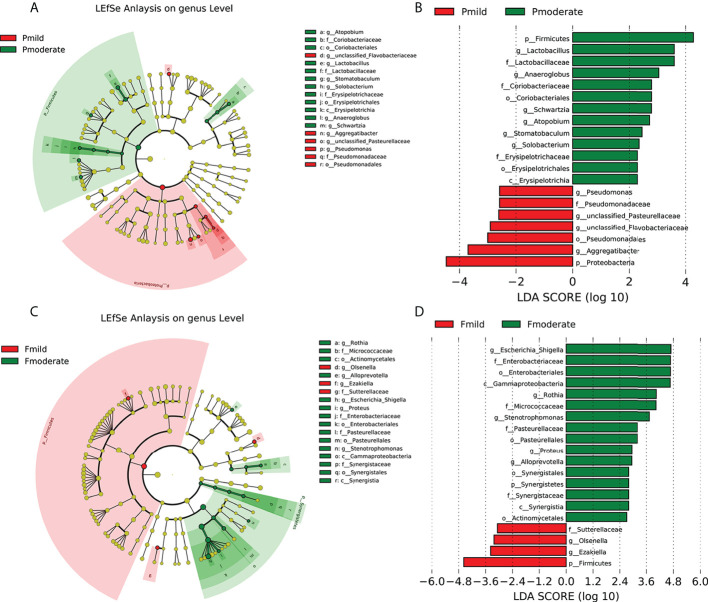
Key taxonomic differences of subgingival plaque and fecal microbiota in patients with mild and moderate AD. Cladogram using LEfSe method indicating the phylogenetic distribution of the subgingival **(A, B)** and fecal **(C, D)** microbiotas. Each circle’s diameter is proportional to the taxon’s abundance. As is shown in the histogram of the LDA scores for differentially abundant taxa, positive LDA scores indicate the enrichment of taxa in moderate AD group (green) relative to the control group (red), and negative LDA scores indicate the depletion of taxa. LDA scores (log10) > 2 and p < 0.05; Pcn, subgingival plaque of normal cognition controls; Fcn, feces of normal cognition controls; Pmild, subgingival plaque of mild AD; Fmild, feces of mild AD; Pmoderate, subgingival plaque of moderate AD; Fmoderate, feces of moderate AD; LEfSe, linear discriminant analysis (LDA) effect size.

To further explore all alterations in the oral and gut microbiotas of the mild AD and moderate AD groups, we used LEfSe analysis (with a LDA score cut off > 2.0) to identify the key taxa responsible for the differences in the compositions of the subgingival and fecal microbiotas between the two groups. In the taxa of subgingival bacteria, 13 taxa were more abundant in the moderate AD group, including the phylum Firmicutes and its corresponding class Erysipelotrichia, the order Erysipelotrichales, the Lactobacillaceae and Erysipelotrichaceae families, the genus *Anaeroglobus*, the genus *Lactobacillus*, the genus *Stomatobaculum*, the genus *Schwartzia*, and the Actinobacteria phylum’s corresponding order Coriobacteriales and family Coriobacteriaceae, the genus *Atopobium*; and the genus *Solobacterium*. The phylum Proteobacteria and its corresponding order Pseudomonadales and family Pseudomonadaceae, the genus *Aggregatibacter*, the genus *Pseudomonas*; and the genus *unclassified_ Pasteurellaceae* were less abundant (**Figures 5A, B**).

In the taxa of fecal microbiotas, the abundance of 16 taxa (the phylum Synergistetes and its corresponding class Synergistia, order Synergistales and family Synergistales, the phylum Proteobacteria and corresponding class Gammaproteobacteria, the order Pasteurellales, the order Enterobacteriales, the family Pasteurellaceae, the family Enterobacteriaceae, the genus *Stenotrophomonas*, the genus *Proteus*, the genus *Escherichia_Shigella*, and the phylum Actinobacteria’s corresponding order Actinomycetales, the family Micrococcaceae, the genus *Rothia*, and the genus *Alloprevotella*) were enriched in the moderate AD group. However, the abundances of the phylum Firmicutes and its corresponding genus *Ezakiella*, the family Sutterellaceae, and the genus *Olsenella*, were depleted (**Figures 5C, D**).

We were able to differentiate seven key functional taxa between the AD and the control groups. The Firmicutes and Proteobacteria phyla were responsible for most differences in the compositions of the subgingival microbiotas between the two groups, while the phylum Firmicutes, and the Proteobacteria’s corresponding genus *Escherichia_Shigella*, the order Enterobacteriales, the family Enterobacteriaceae, and the class Gammaproteobacteria of the fecal microbiotas had the most differential abundances in the fecal bacteria when compared between the AD groups. The key differences in the microbiomes between the mild AD and moderate AD groups showed approximately similar tendency for variation in the relative abundance of the Firmicutes and Proteobacteria phyla and their corresponding taxa in different clinical stages of AD (normal controls, mild and moderate AD). These key taxa may therefore be used as biomarkers for discriminating between the groups of patients.

### Associations between clinical data and altered microbiomes in the mild and moderate AD groups

**Figure 6:**
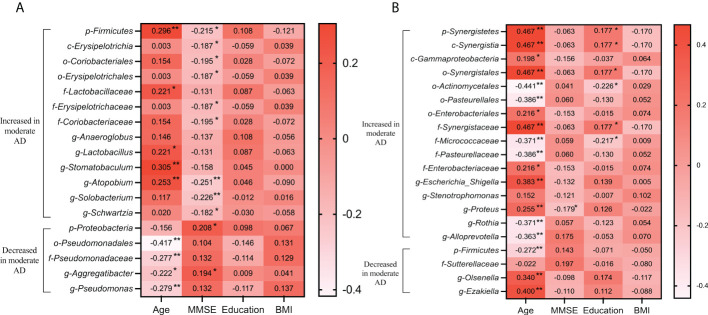
Correlations of altered oral **(A)** and gut **(B)** microbiomes with clinical characteristics in mild and moderate AD groups. Spearman’s rank correlation coefficient (R) and probability (*p*) were used to evaluate statistical importance. R-value showing the correlation between the microbiota and clinical factor, with significant *p*-values indicated. *p <0.05, **p <0.01; BMI, body mass index; MMSE, mini-mental state examination.

To determine the associations between clinical indicators, and the deferential genera of the mild AD and moderate AD groups, we performed correlation analysis using Spearman’s rank correlation (**Figure 6**). In the subgingival microbiotas, the relative abundances in the moderate AD group were higher in bacteria of the phylum Firmicutes and showed positive correlation with age and negative correlation with MMSE scores. However, the relative abundances in the moderate AD group were lower for the phylum Proteobacteria*;* these were positively correlated with MMSE scores. The phylum Proteobacteria’s corresponding order Pseudomonadales, the Family Pseudomonadaceae, the genus *Aggregatibacter* and the genus *Pseudomonas* were negatively correlated with age (**Figure 6A**). In the fecal microbiotas, we found that the genus *Proteus*, from the family of Enterobacteriaceae, were increased in moderate AD and positively correlated with age but negatively associated with MMSE scores (**Figure 6B**) (*p*<0.05).

### Microbial functional dysbiosis in the mild AD, moderate AD, and NC groups

We used PICRUSt to predicte KEGG functional orthologs in level-2 KEGG pathways to identify functional changes in the oral and fecal microbiotas among the three groups. As shown in **Table 2**, in the oral microbiota, the progressively modified orthologs that were more abundant in the mild and moderate AD groups than in the NC group were enriched in membrane transport, carbohydrate metabolism, and signaling molecules and interaction pathways and low in energy metabolism, cellular processes and signaling, folding, sorting and degradation, signal transduction, endocrine system, neurodegenerative diseases and cancers. Importantly, the nine important functional orthologs showed significant alterations in the moderate AD group when compared with healthy subjects.

**Table 2:** PICRUSt-based examination of the oral bacterial functions among the three groups.

	Pcn	Pmild	Pmoderate	*p*-Value	CN vs Mild	CN vs Moderate	Mild vs Moderate
	Median (IQR)	Median (IQR)	Median (IQR)		*p*_1_	*p*_2_	*p*_3_
Membrane Transport	10.584 (9.790-11.394)	10.977 (10.276-11.709)	11.320 (10.349-12.183)	0.013*	–	0.009**	–
Carbohydrate Metabolism	9.089 (8.866-9.441)	9.126 (8.925-9.478)	9.301 (9.072-9.747)	0.015*	–	0.044*	–
Signaling Molecules and Interaction	0.190 (0.177-0.209)	0.202 (0.182-0.233)	0.215 (0.195-0.250)	0.001**	–	0.001**	–
Energy Metabolism	5.866 (5.760-6.061)	5.825 (5.593-5.969)	5.735 (5.564-5.971)	0.027*	–	0.022*	–
Cellular Processes and Signaling	4.048 (3.745-4.408)	4.037 (3.717-4.269)	3.830 (3.579-4.068)	0.003**	–	0.012*	0.023*
Folding, Sorting and Degradation	2.830 (2.748-2.871)	2.792 (2.688-2.848)	2.736 (2.669-2.814)	0.000**	–	0.000**	–
Signal Transduction	1.308 (1.200-1.399)	1.243 (1.149-1.319)	1.195 (1.113-1.305)	0.007**	–	0.005**	–
Endocrine System	0.303 (0.281-0.334)	0.280 (0.257-0.305)	0.275 (0.258-0.309)	0.003**	0.025*	0.003**	–
Neurodegenerative Diseases	0.288 (0.201-0.327)	0.270 (0.183-0.346)	0.226 (0.171-0.303)	0.021*	–	–	–
Cancers	0.103 (0.091-0.118)	0.095 (0.086 -0.104)	0.091 (0.076-0.104)	0.002**	–	0.001**	–

PICRUSt-based examination of the oral bacterial functions among the three groups.

Comparisons between the groups for each KEGG functional category (levels 2) are shown by medians (IQR). P-value was calculated using a Kruskal-Wallis test among the three groups. *p*_1_, *p*_2_, and *p*_3_ adjusted for significance with Bonferroni correction for multiple tests. Pcn, subgingival plaque of normal cognition controls; Pmild, subgingival plaque of mild AD; Pmoderate, subgingival plaque of moderate AD. *p <0.05, ** *p*<0.01.

In the gut microbiota (**Table 3**), seven functional orthologs, including membrane transport, poorly characterized, genetic information processing, metabolism, infectious diseases, and cancers showed a trend towards a progressive increase from NC to mild AD to moderate AD groups. In contrast, amino acid metabolism, energy metabolism, metabolism of cofactors and vitamins, biosynthesis of other secondary metabolites, and the endocrine system, showed a progressively decreasing trend from the NC to the mild AD to the moderate AD groups. All of these were significantly altered in the moderate AD group when compared with the NC group.

**Table 3:** PICRUSt-based examination of the gut bacterial functions among the three groups.

	Fcn	Fmild	Fmoderate	*p*-Value	CN vs Mild	CN vs Moderate	Mild vs Moderate	
	medians (IQR)	medians (IQR)	medians (IQR)		*p*1	*p*2	*p*3	
Membrane Transport	12.017 (11.444-13.145)	13.047 (11.559-13.955)	13.247 (11.928-14.182)	0.044*	–	0.037*	–	
Poorly Characterized	5.045 (4.855-5.318)	5.081 (4.916-5.417)	5.267 (5.010-5.522)	0.007**	–	0.011*	–	
Genetic Information Processing	2.830 (2.683-3.073)	2.886 (2.738-3.165)	3.119 (2.844-3.516)	0.000**	–	0.000**	0.041*	
Metabolism	2.751 (2.499-2.960)	2.645 (2.476-2.876)	2.851 (2.552-3.176)	0.040*	–	–	–	
Infectious Diseases	0.431 (0.381-0.479)	0.434 (0.394-0.525)	0.492 (0.417-0.578)	0.004**	–	0.005**	–	
Neurodegenerative Diseases	0.115 (0.091-0.138)	0.116 (0.081-0.157)	0.132 (0.096-0.170)	0.024*	–	–	–	
Cancers	0.102 (0.092-0.119)	0.106 (0.096-0.12)	0.116 (0.102-0.131)	0.014*	–	0.028*	–	
Amino Acid Metabolism	9.389 (8.970-9.765)	9.297 (8.475-9.667)	9.017 (8.285-9.466)	0.013*	–	0.014*	–	
Energy Metabolism	5.663 (5.443-5.911)	5.593 (5.172-5.986)	5.396 (5.084-5.825)	0.011*	–	0.016*	–	
Metabolism of Cofactors and Vitamins	4.245 (4.369-4.393)	4.183 (4.012-4.393)	4.127 (3.956-4.291)	0.047*	–	–	–	
Biosynthesis of Other Secondary Metabolites	0.948 (0.830-1.04)	0.905 (0.751-0.988)	0.846 (0.715-0.956)	0.004**	–	0.003**	–	
Replication and Repair	8.250 (7.862-8.823)	8.385 (7.486-8.888)	7.920 (7.172-8.552)	0.014*	–	–	0.035*	
Translation	5.063 (4.786-5.534)	5.173 (4.502-5.695)	4.859 (4.289-5.422)	0.032*	–	–	–	
Nucleotide Metabolism	3.813 (3.998-4.048)	3.893 (3.553-4.048)	3.735 (3.452-3.956)	0.047*	–	–	–	
Signal Transduction	1.734 (1.489-1.985)	1.684 (1.397-2.303)	1.984 (1.550-2.375)	0.011*	–	–	0.032*	
Cell Motility	1.679 (1.275-1.932)	1.484 (1.207-1.932)	1.843 (1.509-2.282)	0.034*	–	–	–	
Cell Growth and Death	0.467 (0.433-0.493)	0.476 (0.397-0.515)	0.433 (0.352-0.492)	0.038*	–	–	–	
Endocrine System	0.292 (0.249-0.334)	0.292 (0.233-0.326)	0.263 (0.221-0.308)	0.029*	–	0.033*	–	

PICRUSt-based examination of the fecal bacterial functions among the three groups.

Comparisons between the groups for each KEGG functional category (levels 2) are shown by medians (IQR). p-value was calculated using a Kruskal-Wallis test among the three groups. p_1_, p_2_, and p_3_ adjusted for significance with Bonferroni correction for multiple tests. Fcn, feces of normal cognition controls; Fmild, feces of mild AD; Fmoderate, feces of moderate AD. *p <0.05, **p <0.01.

### Overlapping genera between the oral and gut microbiotas

**Figure 7:**
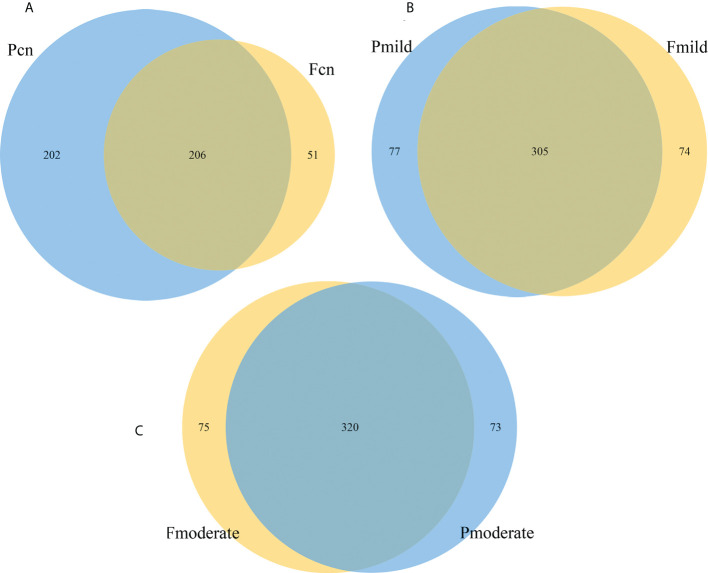
The similarity and overlap with oral and fecal microbiotas. The Venn diagrams illustrate all overlapping genera identified in the oral and gut microbiota in the normal control **(A)**, the mild AD **(B)**, and the moderate AD **(C)** groups. Subgingival and gut microbiomes are colored in blue and yellow nodes, respectively. Pcn, subgingival plaque of normal cognition controls; Fcn, feces of normal cognition controls; Pmild, subgingival plaque of mild AD; Fmild, feces of mild AD; Pmoderate, subgingival plaque of moderate AD; Fmoderate, feces of moderate AD.

Because we characterized gut and oral microbiota samples from the same individuals, we tried to explore the similarity between these microbiotas within patients. Venn diagram analysis showed that in the NC group, the 459 genera fell into three categories: 51 (11.1%) were predominantly fecal, 202 (44.0%) were predominantly oral, and the remaining 206 (44.8%) overlapped between the subgingival and stool samples (**Figure 7A**). In the mild AD group, of the 456 genera, 74 (16.2%) were predominantly fecal, 77 (16.9%) were predominantly oral, and the remaining 305 (66.9%) overlapped between the two samples (**Figure 7B**). The moderate AD group had 468 genera; 75 (16.0%) were predominantly fecal, 73 (15.6%) were predominantly oral, and the remaining 320 (68.4%) overlapped between the subgingival and stool samples (**Figure 7C**). The number of oral-gut overlapping genera showed a trend towards a progressive increase from NC to mild AD to moderate AD groups.

## Discussion

To our knowledge, this is the first study to co-analyze the oral and gut microbiomes of Chinese elderly patients with AD. We found that a tendency for variation in the relative abundance of the Firmicutes and Proteobacteria phyla in different clinical stages of AD (normal controls, mild and moderate AD) and that difference was clearest with the moderate AD group. The key differences in the microbiomes between the mild AD and moderate AD groups showed approximately similar alterations in variation tendency, and the differing bacterial taxa were associated with MMSE scores and age. KEGG analysis showed that the oral and gut functional pathways exhibited significant changes among the three groups. Venn diagram analysis of the number of oral-gut overlapping genera showed a gradual upwards trend from NC to mild AD to moderate AD groups.

### Alterations of oral microbial compositions and gene functions among different AD phases

The gastrointestinal tract begins in the oral cavity where a diverse array of microbes resides. The mouth harbors over 700 bacterial taxa, most residing in the anaerobic environment of the subgingival surface as a biofilm (Arweiler and Netuschil, 2016).The oral microbiota is the second most diverse community in the human body after the gut microbiota (Chen et al., 2017). In this study, we found that the oral abundances of the Firmicutes and Fusobacteria phyla showed a gradual upwards trend from NC to mild to moderate AD groups, while the abundance of the Proteobacteria phylum decreased gradually in the same order. Previous studies have reported results consistent with our current findings. A study by Ling et al. (2015) defined oral supragingival plaque bacterial sequences in healthy 37- to 59-year-old adults that were identical to those that we found with enriched Firmicutes, Actinobacteria, Bacteroidetes, and Fusobacteria phyla. Wu et al. (2021) reported a general increase in Firmicutes, Actinobacteria, and Bacteroidetes, and a reduction in Fusobacteria in the dental plaques of elderly patients with AD when compared with the abundances in controls; in addition, one indicator of aging (Mariat et al., 2009), the ratio of Firmicutes to Bacteroidetes (F/B ratio), was also increased in the patients. In our study, the moderate AD group showed a higher F/B ratio (*p* = 1.098) than the NC group. A higher F/B ratio (>1) reflects an imbalance of the oral Firmicutes and Bacteroidetes in moderate AD patients, thus suggesting an increased systemic inflammatory response (Verdam et al., 2013; Emoto et al., 2016). The cause of these variations may be correlated with the changes in the oral microbiota that occur with age (Wu et al., 2021). We found consistent results in our study, with the patients in the moderate AD group being older and presenting with worse cognitive functions than those in the mild AD and NC groups. The relative abundance of the Firmicutes phylum was positively correlated with age and negatively correlated with MMSE scores, thus suggesting that the increased abundance of the oral Firmicutes phylum in the moderate AD group may be correlated with worse cognitive function; moreover, the positive correlation of the Proteobacteria phylum with MMSE scores is in line with the reduction in abundance with worsening cognitive dysfunction.

KEGG analysis showed that the oral functional pathways in the mild and moderate AD groups were mainly involved in membrane transport and carbohydrate metabolism, which showed a progressively increased prevalence from the mild AD to the moderate AD groups as compared to that in the NC group. These pathways were most abundant in the moderate AD group. The enrichment in membrane transport indicated that oral transmembrane transport was more active in the moderate AD group. With regards to the carbohydrate metabolism pathway, a previous study reported that enhanced carbohydrate activity in the oral microbiota was a contributing factor for the pathogenesis of caries (Peterson et al., 2011). In our study, the moderate AD group was enriched in the oral Firmicutes phylum, bacteria secreting glycoside hydrolases (GHs) involved in the degradation of carbohydrate main chains (White et al., 2014). Thus, an increase of Firmicutes in the oral cavity can increase carbohydrate catabolism in the human body. Moreover, the early colonizing and resident bacteria of the dental plaque biofilms are known to have different types of GHs. We found that the relative abundance of the cariogenic dental pathogens *Lactobacillus* and *Streptococcus* in subgingival plaques was higher in the moderate AD group than in the mild AD and NC groups. *Lactobacillus* is a supragingival saccharolytic bacteria that degrades carbohydrates (such as sugars) into organic acids as well as metabolizing amino acids into acids and ammonia, thus leading to the demineralization of tooth surfaces and the development of caries (Takahashi, 2015). A previous study also reported that the proportions of the cariogenic dental pathogens Lactobacillales and Streptococcaceae were increased in the dental plaques of elderly patients with AD (Emery et al., 2017).

Notably, we found that the abundance of the Fusobacteria phylum was significantly higher in the moderate AD group than in the NC group. In agreement with this finding, Vera et al. (Panzarella et al., 2020) reported a significantly higher Fusobacteria load in patients with AD than in controls. Stein et al. (Sparks Stein et al., 2012) also reported higher antibody levels to Fusobacteria in patients with AD than in controls. Fusobacteria is one of the commonest species in the human gingiva and has a crucial role in the development and progression of periodontal disease (Diaz et al., 2002). In another study, colonization by *Fusobacterium nucleatum* contributed to the development of a more aggressive periodontal disease in an AD group than in controls, with a consequent increase in the number of lost teeth (Panzarella et al., 2020). Accordingly, the patients in the moderate AD group in the current study had a marked reduction in the number of teeth and a higher BOHSE score than those in the mild AD group (**Supplementary Table 1**). These data imply that patients in the moderate AD group presented with worse overall oral health conditions. Pathogenic bacteria in the oral cavity need to cover a shorter distance to invade the brain than gut bacteria in the colon (Cryan et al., 2019). Postmortem brain examinations of patients with AD have reported higher isolation rates for Actinobacteria, *Lactobacillus*, and Streptococcaceae (Emery et al., 2017). These pathogens penetrate the BBB, continuously causing neuroinflammation and promoting neurodegeneration that may lead to cognitive impairment and promote the pathogenesis of AD.

### Alterations of gut microbial compositions and gene functions among different AD phases

Growing evidence indicates that the gut microbiota influences brain function and behavior *via* the microbiota–gut–brain axis (Cryan et al., 2019). In our comparison of gut microbiotas among the three groups, we found that the phylum Firmicutes showed a trend towards a progressive decrease with worsening cognitive decline, while the Proteobacteria phylum increased gradually from the mild AD to the moderate AD groups as compared to its abundance in the NC group. These findings agree with previous results demonstrating lower abundances of Firmicutes and higher abundances of Proteobacteria in patients with AD. In the human gut microbiome, the Firmicutes, Bacteroidetes and Proteobacteria phyla are the most dominant (Kristina et al., 2012). Liu et al. (2019) conducted a Chinese cohort study and found that the relative abundance of Firmicutes was significantly reduced, and the proportion of Proteobacteria was highly enriched, in patients with AD when compared to those in patients with MCI and controls. In addition, similar alterations were observed at the order, class, and family levels of these two phyla. Sheng et al. (2021) revealed that the abundances of the Firmicutes phylum and its corresponding class, order, family, and genus taxa were progressively reduced from NC to SCD and to Cognitive Impairment (CI) groups (the last group showed a significantly decreased abundance of the Firmicutes phylum). Similarly, Hou et al. (2021) showed that the abundances of the phylum Proteobacteria and the *Escherichia-Shigella* and *Ruminococcaceae_UCG_002* genera were increased in patients with AD when compared with those in healthy controls. Both Nagpal et al. (2019) and Zhang et al. (2021) reported the abundances of Proteobacteria and Gammaproteobacteria were higher in participants with MCI when compared with those in healthy controls. A meta-analysis further showed that the abundance of the Proteobacteria phylum increased progressively from an NC group to a MCI and an AD group (Hung et al., 2022).

Based on our results and those of previous studies, the relative abundance of the Firmicutes phylum decreases progressively, while that of the Proteobacteria phylum increases gradually with the progression of the AD spectrum (NC, SCD or MCI, and AD). Thus, these clinical stages have been considered as moderators for alterations in the microbiota (Liu et al., 2019). In discriminating models for the oral and gut microbiomes, alterations of the Firmicutes and Proteobacteria phyla are reversed. In addition, similar alterations have been seen at the order, class, family and genus levels of these two phyla. The novelty of our study is the further discovery of the tendency for variation in the relative abundance of the Firmicutes and Proteobacteria phyla between patients with different AD phases. Our study provides further evidence that human AD is likely to be associated with the progressively diminishing abundance of Firmicutes and the gradually increasing abundance of Proteobacteria in affected patients from healthy individuals to those with moderate AD dementia.

The microbiota may affect neuroinflammation by modulating neurochemical and neurometabolic pathways. In this study, the fecal microbial gene functions related to the metabolism of amino acids, energy, cofactors, and vitamins were significantly lower in the moderate AD group than in the NC group. Binyin Li et al. (2019) reported a similar reduction in functional fecal orthologs reduction in an AD group when compared with that in healthy individuals. Although the specific bacteria responsible for these functional alterations may differ between conditions, alterations in the microbiota of patients with AD may change the balanced metabolism and biosynthesis of fatty acids in the human body (Li et al., 2019). For instance, the presence of the Firmicutes phylum is associated with inflammatory responses, the modulation of metabolic function, and the production of SCFAs (Kumar et al., 2014; Bhat and Kapila, 2017; Welcome, 2019). The reduced abundance of the Firmicutes phylum and its corresponding taxa (e.g., family Acidaminococcaceae, genus *Phascolarctobacterium*, and genus *Clostridium_IV*) may promote the production of toxic metabolites and proinflammatory cytokines and reduce the quantities of beneficial substances, such as SCFAs. The bacteria in the family Acidaminococcaceae and genus *Clostridium_IV* are well-known butyrate producers. The effects of butyrate include a reduction in inflammation, an improvement in oxidative status, and improvements in the integrity of the blood–brain barrier (Canani et al., 2011; Fung et al., 2017); these factors play an important role in the maintenance of brain function in patients with AD. Bacteria in the *Phascolarctobacterium* genus are substantial acetate/propionate producers that might be associated with the metabolic state and mood of the host (Wu et al., 2017). These are all important molecules with regards to maintaining an environmental balance in the intestine and the prevention of inflammation (Kim et al., 2014). Abnormal levels of SCFAs have been proposed to negatively affect disease progression and maintenance, potentially through immune activation and systemic inflammation (Morris et al., 2015) and can lead to damage of the gut epithelial barrier and subsequent blood-brain barrier dysfunction (Welcome, 2019). Neuroinflammation contributes to the progression of neuropathological changes in AD and to the formation of amyloid-β plaques and neurofibrillary tangles (Kauwe et al., 2014; Stella et al., 2014). Interestingly, we also found that microbial infectious diseases increased from the mild AD to the moderate AD groups as compared to those in the NC group. Thus, dysbiosis of the intestinal microflora may contribute to the pathogenesis of AD *via* metabolic pathways. However, more detailed work is now needed to provide specific insights into the effects of these metabolites upon the pathogenesis of AD.

Pro-inflammatory Proteobacteria have been suggested to be a predictor for AD pathogenesis (Hou et al., 2021; Liu et al., 2021). The Proteobacteria are a major phylum of Gram-negative bacteria with lipopolysaccharide (LPS) and are capable of triggering systemic inflammation and the release of pro-inflammatory cytokines after translocation from the gut to the systemic circulation (Cani et al., 2007). Moreover, a recent study reported that the levels of LPS and gram–negative *Escherichia coli* were higher in postmortem brain tissues and blood vessels of patients with AD than those in a control group (Zhan et al., 2016). Notably, we found that the Proteobacteria phylum and its corresponding class Gammaproteobacteria, order Enterobacteriales, family Enterobacteriaceae, and genus *Escherichia_Shigellaa*, were broadly enriched in the moderate AD group when compared with their abundance in the NC group. Furthermore, the abundance of Proteobacteria has been shown to be increased and associated with worsening memory dysfunction levels (Hossain et al., 2019; Jeong et al., 2019). Consistent with these earlier findings, we found that the genus *Proteus*, from the family of Enterobacteriaceae, was increased in moderate AD and negatively associated with MMSE scores. Similarly, Liu et al. (2019) revealed that Gammaproteobacteria, Enterobacteriales, and Enterobacteriaceae were progressively enriched from the prodromal MCI to the AD stages as compared to their abundance in NCs. More importantly, models based on the family of Enterobacteriaceae could clearly distinguish individuals with AD from those with MCI or NCs. Similarly, Hou et al. (2021) showed that the abundances of the Proteobacteria phylum, along with the *Escherichia-Shigella* and *Ruminococcaceae*_UCG_002 genera were increased in AD individuals when compared with their abundance in healthy controls, thus suggesting that the presence of this phylum is a distinctive biomarker to predict the development of AD. Cattaneo et al. (2017) reported that the abundance of fecal *Escherichia/Shigella*, the major genus of Enterobacteriaceae, was increased in patients with AD. These lines of evidence indicate that the Proteobacteria phylum, and its related taxa, might have important roles in the initiation and progression of AD, and suggest the potential for using these taxa as non-invasive biomarkers of cognitive function and to discriminate between mild and moderate AD.

In parallel with other similar studies (Li et al., 2019; Ling et al., 2020), we also found that the abundances of the Actinobacteria and Verrucomicrobia phyla had clearly increased in moderate AD patients, while the abundance of Bacteroidetes had significantly decreased at the phylum level. Similar with the findings of Ling et al. (2020) and Li et al. (2019), we found that the abundances of the lactate-producing genus *Bifidobacterium* of the Actinobacteria was increased in the moderate AD group; this bacterium is known to be highly beneficial in humans (David et al., 2011). Thus, we still need to investigate the therapeutic potential of *Bifidobacterium* in terms of maintaining cognitive function and treating dementia (Ling et al., 2020). Zhuang and colleagues (Zhuang et al., 2018) demonstrated a similar result in Chinese patients with AD. Li et al. (Liu et al., 2019) reported that the genera in the phylum Bacteroides dominated the reduced genera in patients with AD. However, a previous study undertaken in the USA, reported an opposite change in abundance (Vogt et al., 2017), thus suggesting that the composition of Bacteroidetes varies by country (Hung et al., 2022).

### Similarity between the oral and gut microbiotas

Alterations in the bacterial microbiotas in patients with AD may represent candidates for modulating the pathological processes of AD, unless the changes in their abundance is simply caused by AD pathology. The oral–gut–brain axis signaling represents a new avenue in psychiatry that is expected to provide novel targets for the diagnosis and treatment of AD, and for deciphering its pathogenesis (Narengaowa et al., 2021). The oral and gut microbiomes are well segregated due to the presence of an oral–gut barrier, represented by their physical distance and the presence of gastric acid and bile (Tennant et al., 2008; Segata et al., 2012; Ridlon et al., 2014). However, impairment of the oral–gut barrier can allow inter-organ translocation and communication. Aging is associated with oral–gut barrier dysfunction and can increase gut permeability (“leaky gut”) and bacterial translocations (Ulluwishewa et al., 2011; Tran and Greenwood-Van Meerveld, 2013; Iwauchi et al., 2019). Thus, elderly people have fewer functional barriers in the body (Nagpal et al., 2017; Sovran et al., 2019) and have more prevalent oral bacteria transferred to the intestine, thus colonizing these two locations (e.g., *Porphyromonas*, *Fusobacterium*, and *Pseudoramibacter*) to a greater extent than healthy adults (Odamaki et al., 2016; Iwauchi et al., 2019). As a major age-related neurodegenerative disease, intriguingly, we found that cariogenic dental pathogens *Lactobacillus* and *Streptococcus* were enriched in the feces of mild AD and moderate AD subjects when compared with normal cognition controls. Furthermore, we analyzed the oral–gut overlapping microbiota in the post-AD spectrum (NC, mild AD, moderate AD); Venn diagram analysis revealed 206 (44.8%), 305 (66.9%), and 320 (68.4%) genera that overlapped across the three groups (NC, mild AD, moderate AD), respectively; with a trend towards a progressive increase from the NC to the mild AD and to the moderate AD groups. These findings are similar with those in the shared oral and gut microbiome taxa analysis performed at the genus level by Segata et al. (2012). As estimated by the observed OTUs, ACE, and Chao–1 index data, we found that the oral microbial richness showed a progressively decreased prevalence, while the gut microbial richness showed an upwards trend from the NC to the mild AD and to the moderate AD groups. Thus, we hypothesized that patients with moderate AD may have had more mouth-to-gut microbial transmissions than those with mild AD or healthy individuals. The oral microorganisms that enter the gastrointestinal tract, to a certain extent, change the structure of the intestinal microbial community, thus leading to metabolic endotoxins that will further induce inflammation-related changes in various tissues and organs. For example, *P. gingivalis *can enter the intestine by swallowing and then change the composition of the intestinal microbiome and further increase the permeability of the intestinal epithelium (Arimatsu et al., 2014; Feng et al., 2020). Other studies have shown that the administration of *P. gingivalis* can cause changes in the intestinal microbiota, and even induce the upregulation of the mRNA expression of various proinflammatory cytokines (Kato et al., 2018; Ohtsu et al., 2019). There is now increasing evidence to suggest that inflammation holds a pivotal role in the pathogenesis of AD and that immune pathways can potentially comprise primary therapeutic targets (Paouri and Georgopoulos, 2019). Further shotgun metagenomic sequencing should be used to provide a deeper understanding of the oral-gut relationships at the species level (Maki et al., 2021). Schmidt et al. (2019) indicated that 40% of the total identified species were present in both the oral (saliva) and gut communities at the species level in the elderly. In short, the bidirectional crosstalk between these microbiomes may help develop the oral–gut microbiome axis which plays a crucial role in regulating the pathogeneses of various human diseases, including AD.

### α and β diversity

We found that the fecal microbial diversity, as estimated by the Simpson Index, decreased significantly in individuals with moderate AD when compared to healthy elderly adults, although there was no statistically significant difference in terms of oral microbial diversity. Reduced gut microbial diversity in patients with AD has been reported in previous studies (Liu et al., 2019; Sheng et al., 2021). Liu et al. (2019) associated the reduced gut microbial diversity with an increased risk of AD progression. In addition, α diversity analysis showed a significantly higher diversity and lower richness in the oral microbiota than in the gut microbiota. This is consistent with findings reported by Huse et al. (2012) in which stool samples had the highest estimated richness, followed by those of the mouth and other body sites. Consistent with previous reports (Human Microbiome Project, 2012; Russo et al., 2017; Uchino et al., 2021), the compositions of the oral and gut microbiotas were found to differed greatly in the present study and the habitat specificity was high.

### Limitations

Previous studies mainly analyzed the differences in microbial composition between healthy controls and AD groups. In this study, we further subdivided participants with AD into mild AD and moderate AD groups according to their CDR scores. Compared with individuals in the NC group, more alterations were found in the moderate AD group in terms of oral and gut microbial compositions and gene functions than those in the mild AD group. In this study, the MMSE scores of moderate patients with AD averaged 18 and were similar to the MMSE scores ranging from 4 to 19 in most of previous studies; this may account for the more extensive alterations in the microbiotas of subjects with moderate AD. Moreover, in this study, participants in the mild AD group had a larger number of educational years than those in the NC and moderate AD groups. A previous study reported that individuals with higher education levels were more likely to consume colorful fruits and vegetables (Parker and Rhee, 2021) and have a higher cognitive reserve. We hypothesized that the higher education levels of the mild AD group may affect the microbial composition *via* the diet (Coman and Vodnar, 2020; Solch et al., 2022) and owing to their cognitive reserve (Ng et al., 2021); this may explain the smaller difference between the mild AD and NC groups. Further investigations are now needed to explore the microbial alterations in larger cohorts of different patients with AD from different grades with individuals of matching age, gender, and educational level.

Our study has some limitations that need to be considered. First, while we found alterations of the oral and gut microbiota compositions and gene functions between different clinical stages of AD, we used 16S rRNA amplicons rather than metagenomic sequencing, thus limiting our ability to identify specific bacteria at the species level. Second, due to the impact of COVID-19, most patients with severe AD did not visit the memory clinic available during the study. Thus, we only recruited patients with mild and moderate AD. Third, our study was a cross-sectional study; therefore, we are unable to draw conclusions about the causal relationship between changes in the microbiota and AD. To decipher the dynamic interplay between microbiota and AD, a longitudinal follow-up study should include different stages of AD, such as subjective cognitive decline, mild cognitive impairment, and severe AD, the stages that mark the transition from health to AD. Radiomicrobiomics-based approaches (Sheng et al., 2021), characterized by integrating features of brain neuroimaging and information relating to the oral–gut microbiota, may provide novel insights into the potential mechanisms of mouth–gut–brain interactions in the progression of AD.

## Conclusions

To the best of our knowledge, this is the first study to co-analyze alterations in the composition and gene functions of the oral and gut microbiota between different late clinical stages of AD, including mild and moderate AD by comparing variables with healthy elderly individuals in China. In addition, we explored the similarities of the gut and oral microbiotas. Our findings demonstrate that the alterations of the oral and gut microbiomes across the three groups predominantly involved SCFA-producing Firmicutes and inflammation-promoting Proteobacteria. In addition, functional alterations in the oral and fecal microbiotas also suggested that changes in the oral and fecal microbiotas are associated with alterations in the functionality and metabolic activity of patients and may play vital roles in the pathogenesis and development of AD. Further shotgun metagenomic sequencing efforts will provide a deeper understanding of the oral–gut microbiome axis and may provide a potential new therapeutic target for the treatment of late AD.

## Nomenclature

AD, Alzheimer’s disease; mild AD, mild Alzheimer’s disease; moderate AD, moderate Alzheimer’s disease; BMI, body mass index; BOHSE, Kayser-Jones brief oral health status examination; MMSE, mini-mental state examination; NC, normal cognition controls; PCoA, principal coordinate analysis; IQR, interquartile range; Pcn, subgingival plaque of normal cognition controls; Pmild, subgingival plaque of mild AD; Pmoderate, subgingival plaque of moderate AD; Fcn, feces of normal cognition controls; Fmild, feces of mild AD; Fmoderate, feces of moderate AD; LEfSe, linear discriminant analysis (LDA) effect size.

## Data availability statement

The datasets presented in this study can be found in online repositories. The names of the repository/repositories and accession number(s) can be found below: SRA, PRJNA855571.

## Ethics statement

The studies involving human participants were reviewed and approved by The Ethics Committee of Fujian Provincial Hospital. The patients/participants provided their written informed consent to participate in this study.

## Author contributions

LC and XX have contributed equally to this work and share first authorship. LC and XX contributed to the conception and design of the study. XW, BW, and PZ acquired the data. HC and XJ performed the statistical analyses. XX wrote the manuscript. XL and ZH prepared the tables and figures. LC and XX contributed to the interpretation of the results and critical revisions of the manuscript for important intellectual content. HL approved the final version of the manuscript. All authors contributed to the manuscript revision, and they read and approved the submitted version.

## Funding

The Natural Science Foundation of Fujian Province, China (Grant No. 2019J01501) and the Fujian Provincial Health Technology Project of middle-aged and young backbone Talents Training Project, China (Grant No. 2020GGA011) supported this project. The funding sources had no role in the design and execution of the study, the collection, management, analysis, and interpretation of the data, the preparation, review, or approval of the manuscript, or the decision to submit the manuscript for publication.

## Acknowledgments

We thank Yang Xia, from the Department of Clinical Epidemiology, Shengjing Hospital of China Medical University for useful suggestions for our study. We thank all participants for their cooperation and engagement with this study.

## Conflict of interest

The authors declare that the research was conducted in the absence of any commercial or financial relationships that could be construed as a potential conflict of interest.

## Publisher’s note

All claims expressed in this article are solely those of the authors and do not necessarily represent those of their affiliated organizations, or those of the publisher, the editors and the reviewers. Any product that may be evaluated in this article, or claim that may be made by its manufacturer, is not guaranteed or endorsed by the publisher.

## References

[B1] Alzheimer’s Disease International Patterson C (2018). World Alzheimer Report 2018-The state of the art of dementia research: New frontiers. https://www.alzint.org/resource/world-alzheimer-report-2018/ [Accessed September 21, 2018].

[B2] Arimatsu K. Yamada H. Miyazawa H. Minagawa T. Nakajima M. Ryder M. I.. (2014). Oral pathobiont induces systemic inflammation and metabolic changes associated with alteration of gut microbiota. Sci. Rep. 4, 4828. doi: 10.1038/srep04828 24797416 PMC4010932

[B3] Arweiler N. B. Netuschil L. (2016). The oral microbiota. Adv. Exp. Med. Biol. 902, 45–60. doi: 10.1007/978-3-319-31248-4_4 27161350

[B4] Bhat M. I. Kapila R. (2017). Dietary metabolites derived from gut microbiota: Critical modulators of epigenetic changes in mammals. Nutr. Rev. 75 (5), 374–389. doi: 10.1093/nutrit/nux001 28444216

[B5] Bolyen E. Rideout J. R. Dillon M. R. Bokulich N. A. Abnet C. C. Al-Ghalith G. A.. (2019). Reproducible, interactive, scalable and extensible microbiome data science using qiime 2. Nat. Biotechnol. 37 (8), 852–857. doi: 10.1038/s41587-019-0209-9 31341288 PMC7015180

[B6] Bonnechère B. Karamujić-Čomić H. Radjabzadeh D. Ahmad S. Ikram M. A. Hankemeier T.. (2020). The role of the gut microbiome in cognitive function and alzheimer’s disease. Alzheimer's Dementia 16 (Suppl. 4), e043197. doi: 10.1002/alz.043197

[B7] Canani R. B. Costanzo M. D. Leone L. Pedata M. Meli R. Calignano A. (2011). Potential beneficial effects of butyrate in intestinal and extraintestinal diseases. World J. Gastroenterol. 17 (12), 1519–1528. doi: 10.3748/wjg.v17.i12.1519 21472114 PMC3070119

[B8] Cani P. D. Amar J. Iglesias M. A. Poggi M. Knauf C. Bastelica D.. (2007). Metabolic endotoxemia initiates obesity and insulin resistance. Diabetes 56 (7), 1761–1772. doi: 10.2337/db06-1491 17456850

[B9] Cattaneo A. Cattane N. Galluzzi S. Provasi S. Lopizzo N. Festari C.. (2017). Association of brain amyloidosis with pro-inflammatory gut bacterial taxa and peripheral inflammation markers in cognitively impaired elderly. Neurobiol. Aging 49, 60–68. doi: 10.1016/j.neurobiolaging.2016.08.019 27776263

[B10] Chen C. K. Wu Y. T. Chang Y. C. (2017). Association between chronic periodontitis and the risk of alzheimer's disease: A retrospective, population-based, matched-cohort study. Alzheimers Res. Ther. 9 (1), 56. doi: 10.1186/s13195-017-0282-6 28784164 PMC5547465

[B11] Cockburn A. F. De Hlin J. M. Ngan T. Crout R. Boskovic G. De Nvir J.. (2012). High throughput DNA sequencing to detect differences in the subgingival plaque microbiome in elderly subjects with and without dementia. Investig. Genet. 3 (1), 19. doi: 10.1186/2041-2223-3-19 PMC3488532 22998923

[B12] Coman V. Vodnar D. C. (2020). Gut microbiota and old age: Modulating factors and interventions for healthy longevity. Exp. Gerontol 141, 111095. doi: 10.1016/j.exger.2020.111095 32979504 PMC7510636

[B13] Cryan J. F. O'Riordan K. J. Cowan C. S. M. Sandhu K. V. Bastiaanssen T. F. S. Boehme M.. (2019). The microbiota-Gut-Brain axis. Physiol. Rev. 99 (4), 1877–2013. doi: 10.1152/physrev.00018.2018 31460832

[B14] David A. Cameld* L. O. Scholey A. B. Pipingas A. Stough C. (2011). Dairy constituents and neurocognitive health in ageing. Br. J. Nutr. 106 (2), 159–174. doi: 10.1017/S0007114511000158 21338538

[B15] Diaz P. I. Zilm P. S. Rogers A. H. (2002). Fusobacterium nucleatum supports the growth of porphyromonas gingivalis in oxygenated and carbon-Dioxide-Depleted environments. Microbiol. (13500872) 148 (2), 467–472. doi: 10.1099/00221287-148-2-467 11832510

[B16] Dominy S. S. Lynch C. Ermini F. Benedyk M. Marczyk A. Konradi A.. (2019). Porphyromonas gingivalis in alzheimer’s disease brains: Evidence for disease causation and treatment with small-molecule inhibitors. Sci. Adv. 5 (1), eaau3333. doi: 10.1126/sciadv.aau3333 30746447 PMC6357742

[B17] Dubois B. Feldman H. H. Jacova C. Hampel H. Molinuevo J. L. Blennow K.. (2014). Advancing research diagnostic criteria for alzheimer's disease: The iwg-2 criteria. Lancet Neurol. 13 (6), 614–629. doi: 10.1016/s1474-4422(14)70090-0 24849862

[B18] Emery D. C. Shoemark D. K. Batstone T. E. Waterfall C. M. Coghill J. A. Cerajewska T. L.. (2017). 16s rrna next generation sequencing analysis shows bacteria in alzheimer's post-mortem brain. Front. Aging Neurosci. 9. doi: 10.3389/fnagi.2017.00195 PMC5476743 28676754

[B19] Emoto T. Yamashita T. Sasaki N. Hirota Y. Hayashi T. So A.. (2016). Analysis of gut microbiota in coronary artery disease patients: A possible link between gut microbiota and coronary artery disease. J. Atheroscler Thromb. 23 (8), 908–921. doi: 10.5551/jat.32672 26947598 PMC7399299

[B20] Feng Y. K. Wu Q. L. Peng Y. W. Liang F. Y. You H. J. Feng Y. W.. (2020). Oral p. gingivalis impairs gut permeability and mediates immune responses associated with neurodegeneration in Lrrk2 R1441g mice. J. Neuroinflamm. 17 (1), 347. doi: 10.1186/s12974-020-02027-5 PMC7677837 33213462

[B21] Fung T. C. Olson C. A. Hsiao E. Y. (2017). Interactions between the microbiota, immune and nervous systems in health and disease. Nat. Neurosci. 20 (2), 145–155. doi: 10.1038/nn.4476 28092661 PMC6960010

[B22] Guo M. Peng J. Huang X. Xiao L. Huang F. Zuo Z. (2021). Gut microbiome features of Chinese patients newly diagnosed with alzheimer's disease or mild cognitive impairment. J. Alzheimers Dis. 80 (1), 299–310. doi: 10.3233/JAD-201040 33523001

[B23] Haririan H. Andrukhov O. Bertl K. Lettner S. Kierstein S. Moritz A.. (2014). Microbial analysis of subgingival plaque samples compared to that of whole saliva in patients with periodontitis. J. Periodontol 85 (6), 819–828. doi: 10.1902/jop.2013.130306 24144271

[B24] Holmer J. Aho V. Eriksdotter M. Paulin L. Pietiäinen M. Auvinen P.. (2021). Subgingival microbiota in a population with and without cognitive dysfunction. J. Oral. Microbiol. 13 (1), 1854552. doi: 10.1080/20002297.2020.1854552 33537116 PMC7833025

[B25] Honig L. S. Vellas B. Woodward M. Boada M. Siemers E. (2018). Trial of solanezumab for mild dementia due to alzheimer's disease. N. Engl. J. Med. 378 (4), 321. doi: 10.1056/NEJMoa1705971 29365294

[B26] Hossain S. Beydoun M. A. Kuczmarski M. F. Tajuddin S. Evans M. K. Zonderman A. B. (2019). The interplay of diet quality and alzheimer's disease genetic risk score in relation to cognitive performance among urban African americans. Nutrients 11 (9), 2181. doi: 10.3390/nu11092181 PMC6769979 31514322

[B27] Hou M. Xu G. Ran M. Luo W. Wang H. (2021). Apoe-Epsilon4 carrier status and gut microbiota dysbiosis in patients with Alzheimer disease. Front. Neurosci. 15. doi: 10.3389/fnins.2021.619051 PMC7959830 33732104

[B28] Human Microbiome Project C. (2012). Structure, function and diversity of the healthy human microbiome. Nature 486 (7402), 207–214. doi: 10.1038/nature11234 22699609 PMC3564958

[B29] Hung C. C. Chang C. C. Huang C. W. Nouchi R. Cheng C. H. (2022). Gut microbiota in patients with alzheimer's disease spectrum: A systematic review and meta-analysis. Aging (Albany NY) 14 (1), 477–496. doi: 10.18632/aging.203826 35027502 PMC8791218

[B30] Huse S. M. Ye Y. Zhou Y. Fodor A. A. (2012). A core human microbiome as viewed through 16s rrna sequence clusters. PloS One 7 (6), e34242. doi: 10.1371/journal.pone.0034242 22719824 PMC3374614

[B31] International, Alzheimer’s Disease, and McGill University. (2021). World Alzheimer Report 2021: Journey through the Diagnosis of Dementia. https://www.alzint.org/resource/world-alzheimer-report-2021/ [Accessed September 21, 2021].

[B32] Iwauchi M. Horigome A. Ishikawa K. Mikuni A. Nakano M. Jz X.. (2019). Relationship between oral and gut microbiota in elderly people. Immunity Inflammation Dis. 7 (3), 229–236. doi: 10.1002/iid3.266 PMC6688080 31305026

[B33] Jeong M. Y. Jang H. M. Kim D. H. (2019). High-fat diet causes psychiatric disorders in mice by increasing proteobacteria population. Neurosci. Lett. 698, 51–57. doi: 10.1016/j.neulet.2019.01.006 30615977

[B34] Kato T. Yamazaki K. Nakajima M. Date Y. Kikuchi J. Hase K.. (2018). Oral administration of porphyromonas gingivalis alters the gut microbiome and serum metabolome. mSphere 3 (5), e00460–18. doi: 10.1128/mSphere.00460-18 30333180 PMC6193602

[B35] Kauwe J. Bailey M. H. Ridge P. G. Perry R. Wadsworth M. E. Hoyt K. L.. (2014). Genome-wide association study of csf levels of 59 alzheimer's disease candidate proteins: Significant associations with proteins involved in amyloid processing and inflammation. PloS Genet. 10 (10), e1004758. doi: 10.1371/journal.pgen.1004758 25340798 PMC4207667

[B36] Kayser-Jones J. Bird W. F. Paul S. M. Long L. Schell E. S. (1995). An instrument to assess the oral health status of nursing home residents. Gerontologist 35 (6), 814–824. doi: 10.1093/geront/35.6.814 8557208

[B37] Kim C. H. Jeongho P. Myunghoo K. (2014). Gut microbiota-derived short-chain fatty acids T cells and inflammation. Immune Network 14 (6), 277. doi: 10.4110/in.2014.14.6.277 25550694 PMC4275385

[B38] Kristina H. Amira K. Geneviève M. Chou C. J. (2012). Is the gut microbiota a new factor contributing to obesity and its metabolic disorders? J. Obes. 2012 (11), 879151. doi: 10.1155/2012/879151 22315672 PMC3270440

[B39] Kumar H. Lund R. Laiho A. Lundelin K. Ley R. E. Isolauri E.. (2014). Gut microbiota as an epigenetic regulator: Pilot study based on whole-genome methylation analysis. Mbio 5 (6), e02113-14. doi: 10.1128/mBio.02113-14 25516615 PMC4271550

[B40] Li B. He Y. Ma J. Huang P. Du J. Cao L.. (2019). Mild cognitive impairment has similar alterations as alzheimer's disease in gut microbiota. Alzheimers Dement 15 (10), 1357–1366. doi: 10.1016/j.jalz.2019.07.002 31434623

[B41] Ling Z. Liu X. Cheng Y. Jiang X. Jiang H. Wang Y.. (2015). Decreased diversity of the oral microbiota of patients with hepatitis b virus-induced chronic liver disease: A pilot project. Sci. Rep. 5, 17098. doi: 10.1038/srep17098 26606973 PMC4660595

[B42] Ling Z. Zhu M. Yan X. Cheng Y. Shao L. Liu X.. (2020). Structural and functional dysbiosis of fecal microbiota in Chinese patients with alzheimer's disease. Front. Cell Dev. Biol. 8. doi: 10.3389/fcell.2020.634069 PMC7889981 33614635

[B43] Liu P. Jia X. Z. Chen Y. Yu Y. Zhang K. Lin Y. J.. (2021). Gut microbiota interacts with intrinsic brain activity of patients with amnestic mild cognitive impairment. CNS Neurosci. Ther. 27 (2), 163–173. doi: 10.1111/cns.13451 32929861 PMC7816203

[B44] Liu X.-X. Jiao B. Liao X.-X. Guo L.-N. Yuan Z.-H. Wang X.. (2019). Analysis of salivary microbiome in patients with alzheimer’s disease. J. Alzheimer's Dis. 72 (2), 633–640. doi: 10.3233/JAD-190587 31594229

[B45] Liu P. Wu L. Peng G. Han Y. Tang R. Ge J.. (2019). Altered microbiomes distinguish alzheimer's disease from amnestic mild cognitive impairment and health in a Chinese cohort. Brain Behav. Immun. 80, 633–643. doi: 10.1016/j.bbi.2019.05.008 31063846

[B46] Lourida I. Hannon E. Littlejohns T. J. Langa K. M. Hyppönen E. Kuźma E.. (2019). Association of lifestyle and genetic risk with incidence of dementia. JAMA 322 (5), 430–437. doi: 10.1001/jama.2019.9879 31302669 PMC6628594

[B47] Maki K. A. Kazmi N. Barb J. J. Ames N. (2021). The oral and gut bacterial microbiomes: Similarities, differences, and connections. Biol. Res. Nurs. 23 (1), 7–20. doi: 10.1177/1099800420941606 32691605 PMC8822203

[B48] Marasco R. A. (2020). Current and evolving treatment strategies for the Alzheimer disease continuum. Am. J. Managed Care 26 (8 Suppl), S167–SS76. doi: 10.37765/ajmc.2020.88481 32840330

[B49] Mariat D. Firmesse O. Levenez F. Guimaraes V. Sokol H. Dore J.. (2009). The Firmicutes/Bacteroidetes ratio of the human microbiota changes with age. BMC Microbiol. 9, 123. doi: 10.1186/1471-2180-9-123 19508720 PMC2702274

[B50] Miklossy J. (1994). Alzheimer Disease–a spirochetosis? Alzheimer Disease. Springer . p, 41–45. doi: 10.1097/00001756-199307000-00002

[B51] Miklossy J. (2008). Alzheimer's disease – a neurospirochetosis. BMC Proc. 2 (Suppl 1), P43. doi: 10.1186/1753-6561-2-s1-p43

[B52] Morris J. C. (1997). Clinical dementia rating: A reliable and valid diagnostic and staging measure for dementia of the Alzheimer type. Int. Psychogeriatr 9 Suppl 1, 173–176. doi: 10.1017/s1041610297004870 9447441

[B53] Morris M. C. Tangney C. C. Wang Y. Sacks F. M. Bennett D. A. Aggarwal N. T. (2015). Mind diet associated with reduced incidence of alzheimer's disease. Alzheimers Dement 11 (9), 1007–1014. doi: 10.1016/j.jalz.2014.11.009 25681666 PMC4532650

[B54] Na H. S. Jung N.-Y. Choi S.. (2020). Analysis of oral microbiome in chronic periodontitis with Alzheimer’s disease: Pilot study. Research Square [Preprint]. Available at: 10.21203/rs.3.rs-24938/v1 (Accessed May 04, 2020).

[B55] Nagpal R. Mainali R. Ahmadi S. Wang S. Yadav H. (2017). Gut microbiome and aging: Physiological and mechanistic insights. Nutr. Healthy Aging 4 (4), 267. doi: 10.3233/NHA-170030 PMC6004897 29951588

[B56] Nagpal R. Neth B. J. Wang S. Craft S. Yadav H. (2019). Modified Mediterranean-ketogenic diet modulates gut microbiome and short-chain fatty acids in association with alzheimer's disease markers in subjects with mild cognitive impairment. EBioMedicine 47, 529–542. doi: 10.1016/j.ebiom.2019.08.032 31477562 PMC6796564

[B57] Narengaowa Kong W. Lan F. Awan U. F. Qing H. Ni J. (2021). The oral-Gut-Brain axis: The influence of microbes in alzheimer's disease. Front. Cell Neurosci. 15. doi: 10.3389/fncel.2021.633735 PMC8079629 33935651

[B58] Ng T. K. S. Slowey P. D. Beltran D. Ho R. C. M. Kua E. H. Mahendran R. (2021). Effect of mindfulness intervention versus health education program on salivary abeta-42 levels in community-dwelling older adults with mild cognitive impairment: A randomized controlled trial. J. Psychiatr. Res. 136, 619–625. doi: 10.1016/j.jpsychires.2020.10.038 33199051

[B59] O'Bryant S. E. Waring S. C. Cullum C. M. Hall J. Lacritz L. Massman P. J.. (2008). Staging dementia using clinical dementia rating scale sum of boxes scores: A Texas alzheimer's research consortium study. Arch. Neurol. 65 (8), 1091–1095. doi: 10.1001/archneur.65.8.1091 18695059 PMC3409562

[B60] Odamaki T. Kato K. Sugahara H. Hashikura N. Takahashi S. Xiao J. Z.. 2016 Age-related changes in gut microbiota composition from newborn to centenarian: A cross-sectional study. BMC Microbiol. 6(1), 90. doi: 10.1186/s12866-016-0708-5 PMC4879732 27220822

[B61] Ogobuiro I. Gonzales J. Tuma F. (2021). Physiology, gastrointestinal. Statpearls (Treasure Island, FL: StatPearls Publishing). 30725788

[B62] Ohtsu A. Takeuchi Y. Katagiri S. Suda W. Maekawa S. Shiba T.. (2019). Influence of porphyromonas gingivalis in gut microbiota of streptozotocin-induced diabetic mice. Oral. Dis. 25 (3), 868–880. doi: 10.1111/odi.13044 30667148

[B63] Panzarella V. Mauceri R. Baschi R. Maniscalco L. Campisi G. Monastero R. (2020). Oral health status in subjects with amnestic mild cognitive impairment and alzheimer's disease: Data from the zabut aging project. J. Alzheimers Dis. 87 (1), 173–183. doi: 10.3233/JAD-200385 PMC9277678 32508326

[B64] Paouri E. Georgopoulos S. (2019). Systemic and cns inflammation crosstalk: Implications for alzheimer's disease. Curr. Alzheimer Res. 16 (6), 559–574. doi: 10.2174/1567205016666190321154618 30907316

[B65] Parker K. Rhee Y. (2021). Alzheimer's disease warning signs: Gender and education influence modifiable risk factors-a pilot survey study. J. Am. Coll. Nutr. 40 (7), 583–588. doi: 10.1080/07315724.2020.1812451 32970519

[B66] Park S.-Y. Hwang B.-O. Lim M. Ok S.-H. Lee S.-K. Chun K.-S.. (2021). Oral–gut microbiome axis in gastrointestinal disease and cancer. Cancers 13 (9), 2124. doi: 10.3390/cancers13092124 33924899 PMC8125773

[B67] Peterson S. N. Snesrud E. Schork N. J. Bretz W. A. (2011). Dental caries pathogenicity: A genomic and metagenomic perspective. Int. Dent. J. 61 Suppl 1, 11–22. doi: 10.1111/j.1875-595X.2011.00025.x PMC3699854 21726221

[B68] Ridlon J. M. Kang D. J. Hylemon P. B. Bajaj J. S. (2014). Bile acids and the gut microbiome. Curr. Opin. Gastroenterol. 30 (3), 332–338. doi: 10.1097/MOG.0000000000000057 24625896 PMC4215539

[B69] Riviere G. R. Riviere K. Smith K. (2002). Molecular and immunological evidence of oral treponema in the human brain and their association with alzheimer's disease. Oral. Microbiol. Immunol. 17 (2), 113–118. doi: 10.1046/j.0902-0055.2001.00100.x 11929559

[B70] Russo E. Bacci G. Chiellini C. Fagorzi C. Niccolai E. Taddei A.. (2017). Preliminary comparison of oral and intestinal human microbiota in patients with colorectal cancer: A pilot study. Front. Microbiol. 8. doi: 10.3389/fmicb.2017.02699 PMC5770402 29375539

[B71] Said H. S. Suda W. Nakagome S. Chinen H. Oshima K. Kim S.. (2014). Dysbiosis of salivary microbiota in inflammatory bowel disease and its association with oral immunological biomarkers. DNA Res. 21 (1), 15–25. doi: 10.1093/dnares/dst037 24013298 PMC3925391

[B72] Sandhu K. V. Sherwin E. Schellekens H. Stanton C. Dinan T. G. Cryan J. F. (2017). Feeding the microbiota-Gut-Brain axis: Diet, microbiome, and neuropsychiatry. Trans. Res. 179, 223–244. doi: 10.1016/j.trsl.2016.10.002 27832936

[B73] Schmidt T. Hayward M. R. Coelho L. P. Li S. S. Bork P. (2019). Extensive transmission of microbes along the gastrointestinal tract. eLife 8, e42693. doi: 10.7554/eLife.42693 30747106 PMC6424576

[B74] Segata N. Haake S. K. Mannon P. Lemon K. P. Waldron L. Gevers D.. (2012). Composition of the adult digestive tract bacterial microbiome based on seven mouth surfaces, tonsils, throat and stool samples. Genome Biol. 13 (6), R42. doi: 10.1186/gb-2012-13-6-r42 22698087 PMC3446314

[B75] Segata N. Izard J. Waldron L. Gevers… D. (2011). Metagenomic biomarker discovery and explanation. Genome Biol. 12 (6), R60. doi: 10.1186/gb-2011-12-6-r60 21702898 PMC3218848

[B76] Sheng C. Lin L. Lin H. Wang X. Han Y. Liu S. L. (2021). Altered gut microbiota in adults with subjective cognitive decline: The silcode study. J. Alzheimers Dis. 82 (2), 513–526. doi: 10.3233/jad-210259 34024839

[B77] Sochocka M. Donskow-Lysoniewska K. Diniz B. S. Kurpas D. Brzozowska E. Leszek J. (2019). The gut microbiome alterations and inflammation-driven pathogenesis of alzheimer's disease-a critical review. Mol. Neurobiol. 56 (3), 1841–1851. doi: 10.1007/s12035-018-1188-4 29936690 PMC6394610

[B78] Solch R. J. Aigbogun J. O. Voyiadjis A. G. Talkington G. M. Darensbourg R. M. O'Connell S.. (2022). Mediterranean Diet adherence, gut microbiota, and alzheimer's or parkinson's disease risk: A systematic review. J. Neurol. Sci. 434, 120166. doi: 10.1016/j.jns.2022.120166 35144237

[B79] Sovran B. Hugenholtz F. Elderman M. Beek A. Graversen K. Huijskes M.. (2019). Age-associated impairment of the mucus barrier function is associated with profound changes in microbiota and immunity. Sci. Rep 11 (8), 799–805. doi: 10.1038/s41598-018-35228-3 PMC6363726 30723224

[B80] Sparks Stein P. Steffen M. J. Smith C. Jicha G. Ebersole J. L. Abner E.. (2012). Serum antibodies to periodontal pathogens are a risk factor for alzheimer's disease. Alzheimers Dement 8 (3), 196–203. doi: 10.1016/j.jalz.2011.04.006 22546352 PMC3712346

[B81] Stella F. Cominetti M. R. Cancela J. M. Forlenza O. V. Talib L. L. Garuffi M.. (2014). Physical exercise in mci elderly promotes reduction of pro-inflammatory cytokines and improvements on cognition and bdnf peripheral levels. Curr. Alzheimer Res. 11 (8), 799–805. doi: 10.2174/156720501108140910122849. 25212919

[B82] Takahashi N. (2015). Oral Microbiome Metabolism: From “who are they?” to “what are they doing?” J. Dent. Res. 94 (12), 1628–1637. doi: 10.1177/0022034515606045 26377570

[B83] Tennant S. M. Hartland E. L. Phumoonna T. Lyras D. Rood J. I. Robins-Browne R. M.. (2008). Influence of gastric acid on susceptibility to infection with ingested bacterial pathogens. Infection Immun. 76 (2), 639–645. doi: 10.1128/IAI.01138-07 PMC2223456 18025100

[B84] Tran L. Greenwood-Van Meerveld B. (2013). Age-associated remodeling of the intestinal epithelial barrier. J. Gerontol A Biol. Sci. Med. Sci. 68 (9), 1045–1056. doi: 10.1093/gerona/glt106 23873964 PMC3738030

[B85] Uchino Y. Goto Y. Konishi Y. Tanabe K. Toda H. Wada M.. (2021). Colorectal cancer patients have four specific bacterial species in oral and gut microbiota in common-a metagenomic comparison with healthy subjects. Cancers (Basel) 13 (13), 3332. doi: 10.3390/cancers13133332 34283063 PMC8268706

[B86] Ulluwishewa D. Anderson R. C. McNabb W. C. Moughan P. J. Wells J. M. Roy N. C. (2011). Regulation of tight junction permeability by intestinal bacteria and dietary components. J. Nutr. 141 (5), 769–776. doi: 10.3945/jn.110.135657 21430248

[B87] Verdam F. J. Fuentes S. de Jonge C. Zoetendal E. G. Erbil R. Greve J. W.. (2013). Human intestinal microbiota composition is associated with local and systemic inflammation in obesity. Obes. (Silver Spring) 21 (12), E607–E615. doi: 10.1002/oby.20466 23526699

[B88] Vogt N. M. Kerby R. L. Dill-McFarland K. A. Harding S. J. Merluzzi A. P. Johnson S. C.. (2017). Gut microbiome alterations in alzheimer's disease. Sci. Rep. 7 (1), 13537. doi: 10.1038/s41598-017-13601-y 29051531 PMC5648830

[B89] Welcome M. O. (2019). Gut microbiota disorder, gut epithelial and blood–brain barrier dysfunctions in etiopathogenesis of dementia: Molecular mechanisms and signaling pathways. NeuroMolecular Med. 21 (3), 205–226. doi: 10.1007/s12017-019-08547-5 31115795

[B90] White B. A. Lamed R. Bayer E. A. Flint H. J. (2014). Biomass utilization by gut microbiomes. Annu. Rev. Microbiol. 68, 279–296. doi: 10.1146/annurev-micro-092412-155618 25002092

[B91] Wu F. Guo X. Zhang J. Zhang M. Ou Z. Peng Y. (2017). Phascolarctobacterium faecium abundant colonization in human gastrointestinal tract. Exp. Ther. Med. 14 (4), 3122–3126. doi: 10.3892/etm.2017.4878 28912861 PMC5585883

[B92] Wu Y. F. Lee W. F. Salamanca E. Yao W. L. Su J. N. Wang S. Y.. (2021). Oral microbiota changes in elderly patients, an indicator of alzheimer's disease. Int. J. Environ. Res. Public Health 18 (8), 4211. doi: 10.3390/ijerph18084211 33921182 PMC8071516

[B93] Zhang X. Wang Y. Liu W. Wang T. Wang L. Hao L.. (2021). Diet quality, gut microbiota, and micrornas associated with mild cognitive impairment in middle-aged and elderly Chinese population. Am. J. Clin. Nutr. 114 (2), 429–440. doi: 10.1093/ajcn/nqab078 33871591

[B94] Zhan X. Stamova B. Jin L. W. DeCarli C. Phinney B. Sharp F. R. (2016). Gram-negative bacterial molecules associate with Alzheimer disease pathology. Neurology 87 (22), 2324–2332. doi: 10.1212/WNL.0000000000003391 27784770 PMC5135029

[B95] Zhao L. (2020). Alzheimer's disease facts and figures. Alzheimer's Dementia 2020), 16(3). doi: 10.1002/alz.12068 32157811

[B96] Zhuang Z. Q. Shen L. L. Li W. W. Fu X. Zeng F. Gui L.. (2018). Gut microbiota is altered in patients with alzheimer's disease. J. Alzheimers Dis. 63 (4), 1337–1346. doi: 10.3233/JAD-180176 29758946

[B97] Zhu Q. Jiang S. Du G. (2020). Effects of exercise frequency on the gut microbiota in elderly individuals. Microbiologyopen 9 (8), e1053. doi: 10.1002/mbo3.1053 32356611 PMC7424259

