# Supplementary material for: A comparison of the composition and functions of the oral and gut microbiotas in Alzheimer’s patients
Source: Front Cell Infect Microbiol. 2022 Aug 24;12:942460. doi: 10.3389/fcimb.2022.942460 (PMC9448892; doi:10.3389/fcimb.2022.942460)
Supplement: Supplementary file 1 [file Table_1.docx]

Supplementary Material

# Supplementary Tables

**Supplementary Table 1.** General characteristics of participants

| Individual | mild AD  (*n* = 43) | moderate AD  (*n* = 89) | *p*-value |
| --- | --- | --- | --- |
| Age, years  medians (IQR) | 79.00  (73.00–85.00) | 82.00  (75.50–86.50) | 0.029* |
| Gender, M/F | 15/28 | 33/56 | 0.806 |
| BMI, kg/m², medians (IQR) | 22.20  (20.60–23.87) | 22.60  (21.40–24.20) | 0.347 |
| Education, medians (IQR) | 11 (8–13) | 9 (5–12) | 0.320 |
| MMSE score, medians (IQR) | 22 (21–23) | 18 (17–19) | <0.001** |
| Hypertension, *n* (%) | 15 (34.9%) | 41 (46.1%) | 0.840 |
| Diabetes, *n* (%) | 5 (11.6%) | 18 (20.2%) | 0.595 |
| Cerebrovascular disease, *n* (%) | 26 (60.5%) | 74 (83.1%) | - |
| Hypercholesterolemia, *n* (%) | 26 (60.5%) | 74 (83.1%) | - |
| Natural Teeth, medians (IQR) | 20.50  (16.00–28.00) | 18.00  (11.75–25.00) | 0.041* |
| Dentures, medians (IQR) | 0 (0–4) | 0 (0–3) | 0.868 |
| BOHSE score, medians (IQR) | 8.00  (6.75–9.00) | 9.00  (8.00–10.00) | 0.001** |

**Supplementary Table 1.** *p-*values calculated using the *x*^2^ test or Mann-Whitney U tests. **p* <0.05, ***p* <0.01. Abbreviations: M, male; F, female; mild AD, mild Alzheimer’s disease; moderate AD, moderate Alzheimer’s disease; BMI, body mass index; BOHSE, Kayser-Jones brief oral health status examination; MMSE, mini-mental state examination.

**Supplementary Table 2.** The α and β Diversity between oral and gut microbiota

| Diversity Index | Shannon | Simpson | the observed OTUs | Chao–1 | ACE | β Diversity |
| --- | --- | --- | --- | --- | --- | --- |
| Pcn Medians (IQR) | 3.466  (3.150–3.803) | 0.064  (0.051–0.106) | 317.500  (285.000–339.000) | 391.030  (348.744–419.070) | 386.737  (356.122–416.992) | - |
| Fcn Medians (IQR) | 3.103  (2.592–3.415) | 0.109  (0.080–0.193) | 336.000  (317.500–369.000) | 437.481  (408.311–482.793) | 433.070  (405.750–466.906) | - |
| *p*_1_ | 0.002** | 0.012* | 0.150 | 0.000** | 0.000** | 0.001** |
| Pmild Medians (IQR) | 3.469  (3.233–3.693) | 0.067  (0.050–0.088) | 276.000  (245.500–302.500) | 337.385  (302.210–373.978) | 335.109  (304.612–367.373) | - |
| Fmild Medians (IQR) | 2.955  (2.175–3.379) | 0.123  (0.080–0.281) | 402.500  (351.500–449.500) | 520.997  (470.081–581.675) | 521.801  (471.393–588.976) | - |
| *p*_2_ | 0.000** | 0.000** | 0.000** | 0.000** | 0.000** | 0.001** |
| Pmoderate Medians (IQR) | 3.467  (3.195–3.695) | 0.068  (0.051–0.091) | 272.000  (247.750–294.500) | 315.482  (290.037–351.158) | 313.493  (291.819–342.082) | - |
| Fmoderate  Medians (IQR) | 2.561  (2.017–3.179) | 0.208  (0.104–0.373) | 391.000  (350.000–439.000) | 510.404  (465.202–558.520) | 510.435  (467.082–554.959) | - |
| *p*_3_ | 0.000** | 0.000** | 0.000** | 0.000** | 0.000** | 0.001** |

**Supplementary Table 2.** **p*<0.05, ***p* <0.01. Abbreviations: Pcn, subgingival plaque of normal cognition controls; Fcn, feces of normal cognition controls; Pmild, subgingival plaque of mild AD; Fmild, feces of mild AD; Pmoderate, subgingival plaque of moderate AD; Fmoderate, feces of moderate AD. *p*_1_, comparison between healthy control and mild AD; *p2*, comparison between NC and moderate AD; *p*_3_, comparison between mild AD and moderate AD.

# Supplementary Figures

**
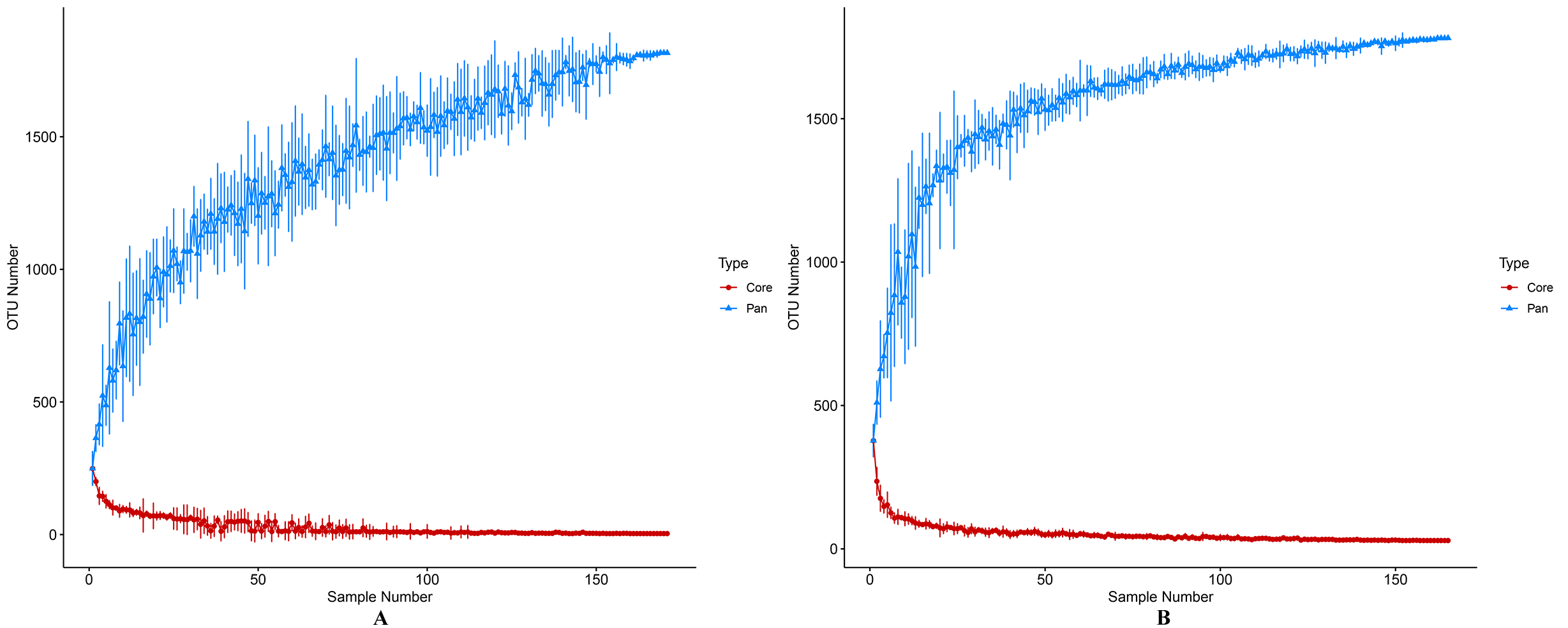
**

**Supplementary Figure 1**. Species accumulation curve of the oral **(A)** and gut **(B)** microbiota. The Pan/Core plot reflects the rate of emergence of new OTUs (new species) under continuous sampling, and the horizontal axis represents the number of samples observed; the vertical axis represents the distribution of the number of common/core OTUs after random sampling by that number. As shown in Figures A and B, when the number of samples reached 150, the species curves were nearly parallel, indicating that when the number of samples exceeded 150, new OUT (new species) no longer increased significantly.
